# Supplementary material for: How outcomes are measured after spontaneous intracerebral hemorrhage: A systematic scoping review
Source: PLoS One. 2021 Jun 30;16(6):e0253964. doi: 10.1371/journal.pone.0253964 (PMC8244847; doi:10.1371/journal.pone.0253964)
Supplement: S1 Table — (DOCX) [file pone.0253964.s002.docx]

SUPPLEMENTAL MATERIAL

How Outcomes are Measured After Spontaneous Intracerebral Hemorrhage: A Systematic Scoping Review of the Current Prospective Literature

S1 Table. References of 395 Included Studies.

| Number | Author(s) | Article Title | Journal Title | Year of Publication |
| --- | --- | --- | --- | --- |
| 1 | Abdullah, J. M.; Husin, A. | Intravascular hypothermia for acute hemorrhagic stroke: A pilot study | Intracerebral Hemorrhage Research: From Bench to Bedside | 2011 |
| 2 | Abdul-Ghaffar, N. U.; el-Sonbaty, M. R.; el-Din Abdul-Baky, M. S.; Marafie, A. A.; al-Said, A. M. | Stroke in Kuwait: a three-year prospective study | Neuroepidemiology | 1997 |
| 3 | Al-Shahi Salman, etc. | Effects of antiplatelet therapy after stroke due to intracerebral haemorrhage (RESTART): a randomised, open-label trial | The Lancet | 2019 |
| 4 | Amir Abdallah | Validation of the Intracerebral Hemorrhage Score in Uganda A Prospective Cohort Study | Stroke | 2018 |
| 5 | Anderson, C.S., etc. | Intensive blood pressure reduction in acute cerebral haemorrhage trial (INTERACT): a randomised pilot trial | Lancet Neurology | 2008 |
| 6 | Anderson, C.S., etc. | Rapid blood-pressure lowering in patients with acute intracerebral hemorrhage | New England Journal of Medicine | 2013 |
| 7 | Arauz, Antonio, etc. | Vascular cognitive disorders and depression after first-ever stroke: the Fogarty-Mexico Stroke Cohort | Cerebrovascular Diseases | 2014 |
| 8 | Asadollahi, S.; Vafaei, A.; Heidari, K. | CT imaging for long-term functional outcome after spontaneous intracerebral haemorrhage: A 3-year follow-up study | Brain Injury | 2016 |
| 9 | Auer, L. M.; Deinsberger, W.; Niederkorn, K.; Gell, G.; Kleinert, R.; Schneider, G.; Holzer, P.; Bone, G.; Mokry, M.; Korner, E. | Endoscopic surgery versus medical treatment for spontaneous intracerebral hematoma: a randomized study | J. Neurosurgery | 1989 |
| 10 | Baharoglu Mi, etc. | Platelet transfusion versus standard care after acute stroke due to spontaneous cerebral haemorrhage associated with antiplatelet therapy (PATCH): a randomised, open-label, phase 3 trial | Lancet | 2016 |
| 11 | Bai, YuLong; Hu, YongShan; Wu, Yi; Zhu, Yulian; He, Qiang; Jiang, CongYu; Sun, LiMin; Fan, WenKe | A prospective, randomized, single-blinded trial on the effect of early rehabilitation on daily activities and motor function of patients with hemorrhagic stroke | Journal of Clinical Neuroscience | 2012 |
| 12 | Baldi, G.; Altomonte, F.; Altomonte, M.; Ghirarduzzi, A.; Brusasco, C.; Parodi, R. C.; Ricciardi, A.; Remollino, V.; Spisni, V.; Saporito, A.; Caiazza, A.; Musso, G.; Cervellin, G.; Lamberti, S.; Buzzalino, M.; De Giorgi, F.; Del Prato, C.; Golinelli, M. P.; Gai, V.; Monsu, R.; Gioffre, M.; Giovanardi, D.; Cattaneo, S.; Frumento, F.; Caporrella, A.; Re, G.; De Iaco, F.; Bologna, G.; Nocenti, F.; Lorenzi, C.; Zoratti, R.; Sciolla, A.; Tiscione, V.; Pastorello, M.; Vandelli, A.; Villa, A.; Zanna, M.; De Palma, A.; Iorio, A. | Intracranial haemorrhage in patients on antithrombotics: clinical presentation and determinants of outcome in a prospective multicentric study in Italian emergency departments | Cerebrovascular Diseases | 2006 |
| 13 | Ballesteros, M. A.; Rubio-Lopez, M. I.; Padilla, A. M. L.; Martin, M. S.; Lopez-Hoyos, M.; Minambres, E. | Serum levels of S100B from jugular bulb as a biomarker of poor prognosis in patients with severe acute brain injury | Journal of the Neurological Sciences | 2018 |
| 14 | Barba, R.; Martinez-Espinosa, S.; Rodriguez-Garcia, E.; Pondal, M.; Vivancos, J.; Del Ser, T. | Poststroke dementia: Clinical features and risk factors | Stroke | 2000 |
| 15 | Barber, M.; Roditi, G.; Stott, D. J.; Langhorne, P. | Poor outcome in primary intracerebral haemorrhage: results of a matched comparison | Post Grad Med J | 2004 |
| 16 | Barton, C. W.; Hemphill, J. C. | Cumulative dose of hypertension predicts outcome in intracranial hemorrhage better than American Heart Association guidelines | Academic Emergency Medicine | 2007 |
| 17 | Bath, P. M. W.; Pathansali, R.; Iddenden, R.; Bath, F. J. | The effect of transdermal glyceryl trinitrate, a nitric oxide donor, on blood pressure and platelet function in acute stroke | Cerebrovascular Diseases | 2001 |
| 18 | Bath, P. M.; Scutt, P.; Anderson, C. S.; Ankolekar, S.; Appleton, J. P.; Berge, E.; Cala, L.; Dixon, M.; England, T. J.; Godolphin, P. J.; Havard, D.; Haywood, L.; Hepburn, T.; Krishnan, K.; Mair, G.; Montgomery, A. A.; Muir, K.; Phillips, S. J.; Pocock, S.; Potter, J.; Price, C. I.; Randall, M.; Robinson, T. G.; Roffe, C.; Rothwell, P. M.; Sandset, E. C.; Sanossian, N.; Saver, J. L.; Shone, A.; Siriwardena, A. N.; Wardlaw, J. M.; Woodhouse, L. J.; Venables, G.; Sprigg, N.; Right, Investigators | Prehospital transdermal glyceryl trinitrate in patients with ultra-acute presumed stroke (RIGHT-2): an ambulance-based, randomised, sham-controlled, blinded, phase 3 trial | Stroke | 2016 |
| 19 | Batjer Hh, Reisch J. S. Allen B. C. Plaizier L. J. Su C. J. | Failure of surgery to improve outcome in hypertensive putaminal hemorrhage. A prospective randomized trial | Archives of Neurology | 1990 |
| 20 | Bauer, Andrew M.; Rasmussen, Peter A.; Bain, Mark D. | Initial Single-Center Technical Experience With the BrainPath System for Acute Intracerebral Hemorrhage Evacuation | Operative neurosurgery | 2017 |
| 21 | Becattini, C.; Franco, L.; Masotti, L.; Nitti, C.; Cattinelli, S.; Cappelli, R.; Manina, G.; Sbrojavacca, R.; Pomero, F.; Agnelli, G. | Clinical management and outcome of major bleeding in patients on treatment with vitamin K antagonists | European journal of internal medicine | 2016 |
| 22 | Bernhardt, J.; Langhorne, P.; Lindley, R. I.; Thrift, A. G.; Ellery, F.; Collier, J.; Churilov, L.; Moodie, M.; Dewey, H.; Donnan, G.; Grp, Avert Trial Collaboration | Efficacy and safety of very early mobilisation within 24 h of stroke onset (AVERT): a randomised controlled trial | Lancet | 2015 |
| 23 | Bhaskar, M. K.; Kumar, R.; Ojha, B.; Singh, S. K.; Verma, N.; Verma, R.; Chandra, A.; Srivastava, C.; Jaiswal, M.; Jaiswal, S.; Huliyappa, H. | A randomized controlled study of operative versus nonoperative treatment for large spontaneous supratentorial intracerebral hemorrhage | Neurology India | 2017 |
| 24 | Bian, Lin; Mao, Lian-Gang; Sun, Yi; Shen, Feng; Chen, Jun-Feng; Liu, Zheng; Zhou, Wei | Serum lipoprotein-associated phospholipase A2 as a promising prognostic biomarker in association with 90-day outcome of acute intracerebral hemorrhage | International Journal of Clinical Chemistry | 2019 |
| 25 | Bilbao, G.; Garibi, J.; Pomposo, I.; Pijoan, J. I.; Carrasco, A.; Catalan, G.; Gonzalez, S. | A prospective study of a series of 356 patients with supratentorial spontaneous intracerebral haematomas treated in a Neurosurgical Department | Acta Neurochirugica | 2005 |
| 26 | Bladin, C. F.; Alexandrov, A. V.; Bellavance, A.; Bornstein, N.; Chambers, B.; Cote, R.; Lebrun, L.; Pirisi, A.; Norris, J. W. | Seizures after stroke: a prospective multicenter study | Archives of Neurology | 2000 |
| 27 | Bobinger, Tobias; Kallmunzer, Bernd; Kopp, Markus; Kurka, Natalia; Arnold, Martin; Hilz, Max-Josef; Huttner, Hagen B.; Schwab, Stefan; Kohrmann, Martin | Prevalence and impact on outcome of electrocardiographic early repolarization patterns among stroke patients: a prospective observational study | Clinical research in cardiology | 2015 |
| 28 | Boru, Ulku Turk; Ozturk, Esat; Tasdemir, Mustafa; Sur, Haydar | Living alone following first-ever stroke: a prospective study in Turkey identifying the risk factors and evaluating their effects | The New Zealand medical journal | 2007 |
| 29 | Bosel J, Schiller P. Hook Y. Andes M. Neumann J. O. Poli S. Amiri H. Schonenberger S. Peng Z. Unterberg A. Hacke W. Steiner T. | Stroke-related early tracheostomy versus prolonged orotracheal intubation in neurocritical care trial (SETPOINT): a randomized pilot trial | Stroke | 2013 |
| 30 | Boysen, G.; Christensen, H. | Stroke severity determines body temperature in acute stroke | Stroke | 2001 |
| 31 | Broessner, Gregor; Beer, Ronny; Lackner, Peter; Helbok, Raimund; Fischer, Marlene; Pfausler, Bettina; Rhorer, Janelle; Kuppers-Tiedt, Lea; Schneider, Dietmar; Schmutzhard, Erich | Prophylactic, endovascularly based, long-term normothermia in ICU patients with severe cerebrovascular disease: bicenter prospective, randomized trial | Stroke | 2009 |
| 32 | Brott, T.; Broderick, J.; Kothari, R.; Barsan, W.; Tomsick, T.; Sauerbeck, L.; Spilker, J.; Duldner, J.; Khoury, J. | Early hemorrhage growth in patients with intracerebral hemorrhage | Stroke | 1997 |
| 33 | Bush, Robin A.; Beaumont, Jennifer L.; Liotta, Eric M.; Maas, Matthew B.; Naidech, Andrew M. | Fever Burden and Health-Related Quality of Life After Intracerebral Hemorrhage | Neurocritical Care | 2018 |
| 34 | Caeiro, L.; Ferro, J. M.; Pinho, E. Melo T.; Canhao, P.; Figueira, M. L. | Post-stroke apathy: An exploratory longitudinal study | Cerebrovascular Diseases | 2013 |
| 35 | Cai, Jing; Li, Jingbo; Chen, Shuda; Meng, Jing; Ren, Reng; Li, Min | Early post-haemorrhagic stroke testosterone and oestradiol levels and long-term risk of death | Brain injury | 2017 |
| 36 | Camps-Renom, P.; Alejaldre-Monforte, A.; Delgado-Mederos, R.; Martinez-Domeno, A.; Prats-Sanchez, L.; Pascual-Goni, E.; Marti-Fabregas, J. | Does prior antiplatelet therapy influence hematoma volume and hematoma growth following intracerebral hemorrhage? Results from a prospective study and a meta-analysis | European journal of neurology | 2017 |
| 37 | Camps-Renom, P.; Mendez, J.; Granell, E.; Casoni, F.; Prats-Sanchez, L.; Martinez-Domeno, A.; Guisado-Alonso, D.; Marti-Fabregas, J.; Delgado-Mederos, R. | Transcranial Duplex Sonography Predicts Outcome following an Intracerebral Hemorrhage | AJNR. American journal of neuroradiology | 2017 |
| 38 | Caprio, Fan Z.; Maas, Matthew B.; Rosenberg, Neil F.; Kosteva, Adam R.; Bernstein, Richard A.; Alberts, Mark J.; Prabhakaran, Shyam; Naidech, Andrew M | Leukoaraiosis on magnetic resonance imaging correlates with worse outcomes after spontaneous intracerebral hemorrhage | Stroke | 2013 |
| 39 | Castellanos, M.; Leira, R.; Tejada, J.; Gil-Peralta, A.; Davalos, A.; Castillo, J.; Stroke Project Cerebrovascular, Dis | Predictors of good outcome in medium to large spontaneous supratentorial intracerebral haemorrhages | Journal of Neurology Neurosurgery and Psychiatry | 2005 |
| 40 | Cereda, Carlo W.; George, Paul M.; Pelloni, Lorenzo S.; Gandolfi-Decristophoris, Paola; Mlynash, Michael; Biancon Montaperto, Lucia; Limoni, Costanzo; Stojanova, Vesna; Malacrida, Roberto; Stadler, Claudio; Bassetti, Claudio L. | Beneficial effects of a semi-intensive stroke unit are beyond the monitor | Cerebrovascular Diseases | 2015 |
| 41 | Chamorro, A.; Horcajada, J. P.; Obach, V.; Vargas, M.; Revilla, M.; Torres, F.; Cervera, A.; Planas, A. M.; Mensa, J. | The Early Systemic Prophylaxis of Infection After Stroke study: a randomized clinical trial | Stroke | 2005 |
| 42 | Chang, J. J.; Kim-Tenser, M.; Emanuel, B. A.; Jones, G. M.; Chapple, K.; Alikhani, A.; Sanossian, N.; Mack, W. J.; Tsivgoulis, G.; Alexandrov, A. V.; Pourmotabbed, T. | Minocycline and matrix metalloproteinase inhibition in acute intracerebral hemorrhage: a pilot study | European Journal of Neurology | 2017 |
| 43 | Chen Cc, Chen X. Li T. C. Lin H. L. Chu Y. T. Lee H. C. Cheng Y. K. Chen D. C. Tsai S. C. Cho D. Y. Hsieh C. L. | PG2 for patients with acute spontaneous intracerebral hemorrhage: a double-blind, randomized, placebo-controlled study | Scientific Reports | 2017 |
| 44 | Chen C-C, Lee H. C. Chang J. H. Chen S. S. Li T. C. Tsai C. H. Cho D. Y. Hsieh C. L. | Chinese herb Astragalus membranaceus enhances recovery of hemorrhagic stroke: double-blind, placebo-controlled, randomized study | Evidence-based complementary and alternative medicine | 2012 |
| 45 | Chen, Bin; Shen, Jia; Zheng, Guan-Rong; Qiu, Shen-Zhong; Yin, Huai-Ming; Mao, Wei; Wang, Hong-Xiang; Gao, Jian-Bo | Serum cyclophilin A concentrations and prognosis of acute intracerebral hemorrhage | International Journal of Clinical Chemistry | 2018 |
| 46 | Chen, C. C.; Liu, C. L.; Tung, Y. N.; Lee, H. C.; Chuang, H. C.; Lin, S. Z.; Cho, D. Y. | Endoscopic surgery for Intraventricular Hemorrhage (IVH) caused by thalamic hemorrhage: Comparisons of endoscopic surgery and External Ventricular Drainage (EVD) surgery | World Neurosurgery | 2011 |
| 47 | Chen, Chih-Wei; Wu, En-Hsuan; Huang, Judy; Chang, Wen-Tsan; Ao, Kam-Hou; Cheng, Tain-Junn; Yang, Wuyang | Dynamic evolution of D-dimer level in cerebrospinal fluid predicts poor outcome in patients with spontaneous intracerebral hemorrhage combined with intraventricular hemorrhage | Journal of Clinical Neuroscience | 2016 |
| 48 | Chen, Yi-Chun; Chen, Chiung-Mei; Liu, Jun-Liang; Chen, Sien-Tsong; Cheng, Mei-Ling; Chiu, Daniel Tsun-Yee | Oxidative markers in spontaneous intracerebral hemorrhage: leukocyte 8-hydroxy-2'-deoxyguanosine as an independent predictor of the 30-day outcome | Journal of Neurosurgery | 2011 |
| 49 | Chen, Ying; Xu, Weihai; Wang, Lijuan; Yin, Xiaoming; Cao, Jie; Deng, Fang; Xing, Yingqi; Feng, Jiachun | Transcranial Doppler combined with quantitative EEG brain function monitoring and outcome prediction in patients with severe acute intracerebral hemorrhage | Critical Care | 2018 |
| 50 | Cheng, C. Y.; Hsu, C. Y.; Huang, Y. C.; Tsai, Y. H.; Hsu, H. T.; Yang, W. H.; Lin, H. C.; Wang, T. C.; Cheng, W. C.; Yang, J. T.; Lee, T. C.; Lee, M. H. | Motor outcome of deep intracerebral haemorrhage in diffusion tensor imaging: comparison of data from different locations along the corticospinal tract | Neurological Research | 2015 |
| 51 | Cheng, X.; Zhang, L.; Xie, N. C.; Ma, Y. Q.; Lian, Y. J. | High Plasma Levels of d-Dimer Are Independently Associated with a Heightened Risk of Deep Vein Thrombosis in Patients with Intracerebral Hemorrhage | Molecular Neurobiology | 2016 |
| 52 | Chiu, Chun-Cheng; Li, Yi-Nong; Lin, Leng-Jye; Hsiao, Cheng-Ting; Hsiao, Kuang-Yu; Chen, I. Chuan | Serum D-dimer as a predictor of mortality in patients with acute spontaneous intracerebral hemorrhage | Journal of Clinical Neuroscience | 2012 |
| 53 | Cho, Der-Yang; Chen, Chun-Chung; Chang, Cheng-Siu; Lee, Wen-Yuan; Tso, Melain | Endoscopic surgery for spontaneous basal ganglia hemorrhage: comparing endoscopic surgery, stereotactic aspiration, and craniotomy in noncomatose patients | Surgical neurology | 2006 |
| 54 | Cho, Der-Yang; Chen, Chun-Chung; Lee, Wen-Yuan; Lee, Han-Chung; Ho, Li-Hwei | A new Modified Intracerebral Hemorrhage score for treatment decisions in basal ganglia hemorrhage--a randomized trial | Critical care medicine | 2008 |
| 55 | Chu, Heling; Huang, Chuyi; Dong, Jing; Yang, Xiaobo; Xiang, Jun; Dong, Qiang; Tang, Yuping | Lactate Dehydrogenase Predicts Early Hematoma Expansion and Poor Outcomes in Intracerebral Hemorrhage Patients | Translational stroke research | 2019 |
| 56 | Cortes-Vicente, Elena; Guisado-Alonso, Daniel; Delgado-Mederos, Raquel; Camps-Renom, Pol; Prats-Sanchez, Luis; Martinez-Domeno, Alejandro; Marti-Fabregas, Joan | Frequency, Risk Factors, and Prognosis of Dehydration in Acute Stroke | Frontiers in neurology | 2019 |
| 57 | Dai, H. Y.; Yang, Y. S.; Guo, F. Q.; Liu, J.; Yu, N. W. | Effects of nimodipine on nervous functions, ability of daily life and plasma neuron-specific enolase of patients with hypertensive cerebral hemorrhage | Chinese Journal of Clinical Rehabilitation | 2005 |
| 58 | D'Amore, Cataldo; Paciaroni, Maurizio; Silvestrelli, Giorgio; Agnelli, Giancarlo; Santucci, Pamela; Lanari, Alessia; Alberti, Andrea; Venti, Michele; Acciarresi, Monica; Caso, Valeria | Severity of acute intracerebral haemorrhage, elderly age and atrial fibrillation: independent predictors of poor outcome at three months | European journal of internal medicine | 2013 |
| 59 | Daverat, P.; Castel, J. P.; Dartigues, J. F.; Orgogozo, J. M. | Death and functional outcome after spontaneous intracerebral hemorrhage. A prospective study of 166 cases using multivariate analysis | Stroke | 1991 |
| 60 | de Ridder, Inger R.; den Hertog, Heleen M.; van Gemert, H. Maarten A.; Schreuder, A. H. C. M. L. Tobien; Ruitenberg, Annemieke; Maasland, E. Lisette; Saxena, Ritu; van Tuijl, Jordie H.; Jansen, Ben P. W.; Van den Berg-Vos, Renske M.; Vermeij, Frederique; Koudstaal, Peter J.; Kappelle, L. Jaap; Algra, Ale; van der Worp, H. Bart; Dippel, Diederik W. J.; Trial, Organization | PAIS 2 (Paracetamol [Acetaminophen] in Stroke 2): Results of a Randomized, Double-Blind Placebo-Controlled Clinical Trial | Stroke | 2017 |
| 61 | Del Brutto, Oscar H.; Campos, Xavier | Validation of intracerebral hemorrhage scores for patients with pontine hemorrhage | Neurology | 2004 |
| 62 | Delgado, P.; Alvarez-Sabin, J.; Abilleira, S.; Santamarina, E.; Purroy, F.; Arenillas, J. F.; Molina, C. A.; Fernandez-Cadenas, I.; Rosell, A.; Montaner, J. | Plasma d-dimer predicts poor outcome after acute intracerebral hemorrhage | Neurology | 2006 |
| 63 | Demchuk Am, Dowlatshahi D. Rodriguez-Luna D. Molina C. A. Blas Y. S. Dzialowski I. Kobayashi A. Boulanger J. M. Lum C. Gubitz G. Padma V. Roy J. Kase C. S. Kosior J. Bhatia R. Tymchuk S. Subramaniam S. Gladstone D. J. Hill M. D. Aviv R. I. | Prediction of haematoma growth and outcome in patients with intracerebral haemorrhage using the CT-angiography spot sign (PREDICT): a prospective observational study | Lancet Neurology | 2012 |
| 64 | den Hertog Hm, van der Worp H. B. van Gemert H. M. Algra A. Kappelle L. J. van Gijn J. Koudstaal P. J. Dippel D. W. Pais Investigators | The Paracetamol (Acetaminophen) In Stroke (PAIS) trial: a multicentre, randomised, placebo-controlled, phase III trial | Lancet Neurology | 2009 |
| 65 | Deogaonkar, Anupa; De Georgia, Michael; Bae, Charles; Abou-Chebl, Alex; Andrefsky, John | Fever is associated with third ventricular shift after intracerebral hemorrhage: pathophysiologic implications | Neurology India | 2005 |
| 66 | Desmettre, T.; Dehours, E.; Samama, C. M.; Jhundoo, S.; Pujeau, F.; Guillaudin, C.; Hecquart, C.; Clerson, P.; Crave, J. C.; Jaussaud, R. | Reversal of Vitamin K Antagonist (VKA) effect in patients with severe bleeding: a French multicenter observational study (Optiplex) assessing the use of Prothrombin Complex Concentrate (PCC) in current clinical practice | Critical care | 2012 |
| 67 | Dhiman, Deepika; Mahajan, Sanjay K.; Sharma, Sudhir; Raina, Rajiv | The Evolving Pattern and Outcome of Stroke at Moderate Altitude | Journal of neurosciences in rural practice | 2018 |
| 68 | Di Napoli, M.; Godoy, D. A.; Campi, V.; Del Valle, M.; Pinero, G.; Mirofsky, M.; Popa-Wagner, A.; Masotti, L.; Papa, F.; Rabinstein, A. A. | C-reactive protein level measurement improves mortality prediction when added to the spontaneous intracerebral hemorrhage score | Stroke | 2011 |
| 69 | Di Napoli, Mario; Godoy, Daniel Agustin; Campi, Veronica; Masotti, Luca; Smith, Craig J.; Parry Jones, Adrian R.; Hopkins, Stephen J.; Slevin, Mark; Papa, Francesca; Mogoanta, Laurentiu; Pirici, Daniel; Popa Wagner, Aurel | C-reactive protein in intracerebral hemorrhage: time course, tissue localization, and prognosis | Neurology | 2012 |
| 70 | Donauer, E.; Loew, F.; Faubert, C.; Alesch, F.; Schaan, M. | Prognostic factors in the treatment of cerebellar haemorrhage | Acta neurochirurgica | 1994 |
| 71 | Dong, Xiao-Qiao; Yu, Wen-Hua; Zhu, Qiang; Cheng, Zhen-Yu; Chen, Yi-Hua; Lin, Xiao-Feng; Ten, Xian-Lin; Tang, Xiao-Bing; Chen, Juan | Changes in plasma thrombospondin-1 concentrations following acute intracerebral hemorrhage | Clinica chimica acta | 2015 |
| 72 | El Hachioui, Lingsma, van de Sandt-Koenderman, Dippel, Koudstaal, Visch-Brink | Long-term prognosis of aphasia after stroke | Journal of Neurology Neurosurgery and Psychiatry | 2013 |
| 73 | El-Sheikh | Stroke Mortality: Predictive Value of Simple Laboratory Tests and APACHE III Scoring System | Egyptian Journal of Neurology, Psychiatry and Neurosurgery | 2010 |
| 74 | Fadel, El-Seidy, Abo-El-Safa, Khalil, Mohamed, Fayed, Mourad, El-Bendary | Prognostic Value of Apolipoprotein E Genotyping in Primary Intracerebral Hemorrhage | Egyptian Journal of Neurology, Psychiatry and Neurosurgery | 2012 |
| 75 | Falcone, Biffi, Devan, Brouwers, Anderson, Valant, Ayres, Schwab, Rost, Goldstein, Viswanathan, Greenberg | Burden of blood pressure-related alleles is associated with larger hematoma volume and worse outcome in intracerebral hemorrhage | Stroke | 2013 |
| 76 | Fan, Chen, Huang, Yen, How, Huang | Interhospital transfer neurological deterioration in patients with spontaneous intracerebral haemorrhage: incidence and risk factors | Postgraduate Medical Journal | 2017 |
| 77 | Femi and Mansur | Factors associated with death and predictors of one-month mortality from stroke in Kano, Northwestern Nigeria | Journal of Neurosciences in Rural Practice | 2013 |
| 78 | Feng, He, Liu, Yang, Wang | Endoscope-Assisted Keyhole Technique for Hypertensive Cerebral Hemorrhage in Elderly Patients: A Randomized Controlled Study in 184 Patients | Turkish Neurosurgery | 2016 |
| 79 | Feng, Lian, Li, Wang | ADVERSE EFFECTS OF WHITE MATTER LESIONS ON LONG-TERM PROGNOSIS OF INTRACEREBRAL HEMORRHAGE | Acta Medica Mediterranea | 2019 |
| 80 | Ferrete-Araujo, Egea-Guerrero, Vilches-Arenas, Godoy, Murillo-Cabezas | Predictors of mortality and poor functional outcome in severe spontaneous intracerebral hemorrhage: a prospective observational study | Medicina Intensiva | 2015 |
| 81 | Ferrete-Araujo, Rodriguez-Rodriguez, Egea-Guerrero, Vilches-Arenas, Godoy, Murillo-Cabezas | Brain Injury Biomarker Behavior in Spontaneous Intracerebral Hemorrhage | World Neurosurgery | 2019 |
| 82 | Finocchi, Balestrino, Malfatto, Mancardi, Serrati, Gandolfo | National Institutes of Health Stroke Scale in patients with primary intracerebral hemorrhage | Neurological Sciences | 2018 |
| 83 | Fischer, Katan, Morgenthaler, Seiler, Muller, Lackner, Errath, Helbok, Pfausler, Beer, Schmutzhard, Broessner | The prognostic value of midregional proatrial natriuretic peptide in patients with hemorrhagic stroke | Cerebrovascular Diseases | 2014 |
| 84 | Foerch, C.; Kessler, K. R.; Steckel, D. A.; Steinmetz, H.; Sitzer, M. | Survival and quality of life outcome after mechanical ventilation in elderly stroke patients | Journal of neurology, neurosurgery, and psychiatry | 2004 |
| 85 | Ford Ga, Bhakta B. B. Cozens A. Hartley S. Holloway I. Meads D. Pearn J. Ruddock S. Sackley C. M. Saloniki E. C. Santorelli G. Walker M. F. Farrin A. J. | Safety and efficacy of co-careldopa as an add-on therapy to occupational and physical therapy in patients after stroke (DARS): a randomised, double-blind, placebo-controlled trial | The lancet neurology | 2019 |
| 86 | Forti, Paola; Maioli, Fabiola; Arnone, Giorgia; Nativio, Valeria; Zagnoni, Silvia; Riva, Letizia; Pedone, Chiara; Pirazzoli, Gian Luca; Coveri, Maura; Zoli, Marco; Di Pasquale, Giuseppe; Procaccianti, Gaetano | Mortality after admission to stroke unit for intracerebral hemorrhage: effect of age 80 and older and multimorbidity | Journal of the American Geriatrics Society | 2015 |
| 87 | Franke, C. L.; van Swieten, J. C.; Algra, A.; van Gijn, J. | Prognostic factors in patients with intracerebral haematoma | Journal of neurology, neurosurgery, and psychiatry | 1992 |
| 88 | Frontera, J. A.; Gordon, E.; Zach, V.; Jovine, M.; Uchino, K.; Hussain, M. S.; Aledort, L. | Reversal of Coagulopathy Using Prothrombin Complex Concentrates is Associated with Improved Outcome Compared to Fresh Frozen Plasma in Warfarin-Associated Intracranial Hemorrhage | Neurocritical Care | 2014 |
| 89 | Fu Y, Hao J. Zhang N. Ren L. Sun N. Li Y. J. Yan Y. Huang D. Yu C. Shi F. D. | Fingolimod for the treatment of intracerebral hemorrhage: a 2-arm proof-of-concept study | JAMA neurology | 2014 |
| 90 | Fu, F.; Sun, S.; Liu, L.; Gu, H.; Su, Y.; Li, Y. | Iodine sign as a novel predictor of hematoma expansion and poor outcomes in primary intracerebral hemorrhage patients | Stroke | 2018 |
| 91 | Furlan, Natalia Eduarda; Bazan, Silmeia Garcia Zanati; Braga, Gabriel Pereira; Castro, Meire Cristina Novelli E.; Franco, Roberto Jorge da Silva; Gut, Ana Lucia; Bazan, Rodrigo; Martin, Luis Cuadrado | Association between blood pressure and acute phase stroke case fatality rate: a prospective cohort study | Arquivos de neuro-psiquiatria | 2018 |
| 92 | Galanth, Sophie; Tressieres, Benoit; Lannuzel, Annie; Foucan, Patrick; Alecu, Cosmin | Factors influencing prognosis and functional outcome one year after a first-time stroke in a Caribbean population | Archives of physical medicine and rehabilitation | 2014 |
| 93 | Gao L, Zhao H. Liu Q. Song J. Xu C. Liu P. Gong W. Wang R. Liu K. J. Luo Y. | Improvement of hematoma absorption and neurological function in patients with acute intracerebral hemorrhage treated with Xueshuantong | Journal of the neurological sciences | 2012 |
| 94 | Garg, R. K.; Liebling, S. M.; Maas, M. B.; Nemeth, A. J.; Russell, E. J.; Naidech, A. M. | Blood Pressure Reduction, Decreased Diffusion on MRI, and Outcomes After Intracerebral Hemorrhage | Stroke | 2012 |
| 95 | Ge, Chunyan; Zhao, Wangmiao; Guo, Hong; Sun, Zhaosheng; Zhang, Wanzeng; Li, Xiaowei; Yang, Xuehui; Zhang, Jinrong; Wang, Dongxin; Xiang, Yi; Mao, Jianhui; Zhang, Wenchao; Guo, Hao; Zhang, Yazhao; Chen, Jianchao | Comparison of the clinical efficacy of craniotomy and craniopuncture therapy for the early stage of moderate volume spontaneous intracerebral haemorrhage in basal ganglia: Using the CTA spot sign as an entry criterion | Clinical neurology and neurosurgery | 2018 |
| 96 | Geurts, M.; de Kort, F. A. S.; de Kort, P. L. M.; van Tuijl, J. H.; van Thiel, Gjmw; Kappelle, L. J.; van der Worp, H. B. | Treatment restrictions in patients with severe stroke are associated with an increased risk of death | European Stroke Journal | 2017 |
| 97 | Ghani, A. R. I.; John, J. T. K.; Idris, Z.; Ghazali, M. M.; Murshid, N. L.; Musa, K. I. | Functional outcome at 6 months in surgical ttreatment of spontaneous supratentorial intracerebral haemorrhage (SICH) | Malaysian Journal of Medical Sciences | 2008 |
| 98 | Godoy, Daniel A.; Pinero, Gustavo R.; Svampa, Silvana; Papa, Francesca; Di Napoli, Mario | Hyperglycemia and short-term outcome in patients with spontaneous intracerebral hemorrhage | Neurocritical Care | 2008 |
| 99 | Godoy, Daniel Agustin; Pinero, Gustavo; Di Napoli, Mario | Predicting mortality in spontaneous intracerebral hemorrhage: can modification to original score improve the prediction? | Stroke | 2006 |
| 100 | Gong, F. T.; Yu, L. P.; Gong, Y. H.; Zhang, Y. X.; Wang, Z. G.; Yan, C. Z. | Blood pressure control in ultra-early basal ganglia intracerebral hemorrhage | European Review for Medical and Pharmacological Sciences | 2015 |
| 101 | Goswami, R. P.; Karmakar, P. S.; Ghosh, A. | Early seizures in first-ever acute stroke patients in India: incidence, predictive factors and impact on early outcome | European Journal of Neurology | 2012 |
| 102 | Goya, Y.; Shibazaki, K.; Sakai, K.; Aoki, J.; Uemura, J.; Saji, N.; Isa, K.; Ohya, Y.; Kimura, K. | Brain natriuretic peptide upon admission as a biological marker of short-term mortality after intracerebral hemorrhage | European neurology | 2014 |
| 103 | Graffagnino, Carmelo; Bergese, Sergio; Love, James; Schneider, Dietmar; Lazaridis, Christos; LaPointe, Marc; Lee, Kiwon; Lynch, Gwendolyn; Hu, Ming-yi; Williams, Gregory C. | Clevidipine rapidly and safely reduces blood pressure in acute intracerebral hemorrhage: the ACCELERATE trial | Cerebrovascular diseases | 2013 |
| 104 | Gu, S. J.; Lu, M.; Xuan, H. F.; Chen, X. Z.; Dong, W. F.; Yan, X. F.; Si, Y.; Gao, G. L.; Hu, D. X.; Miao, J. Q. | Predictive value of serum caspase-cleaved cytokeratin-18 concentrations after acute intracerebral hemorrhage | Clinica Chimica Acta | 2016 |
| 105 | Gupta, Abbot, Srinath, Tewari, Gupta, Gorthi, Narayanan, Totlani, Serohi, Annadure | A randomized trial of safety and efficacy of lowering mean arterial pressurein acute spontaneous intracerebral haemorrhage | Annals of indian academy of neurology | 2016 |
| 106 | Gupta, Mani; Verma, Rajesh; Parihar, Anit; Garg, Ravindra K.; Singh, Maneesh K.; Malhotra, Hardeep S. | Perihematomal edema as predictor of outcome in spontaneous intracerebral hemorrhage | Journal of neurosciences in rural practice | 2014 |
| 107 | Hamel, Goldman, Teno, Lynn, Davis, Harrell, Connors, Califf, Kussin, Bellamy | Identification of comatose patients at high risk for death or severe disability -- SUPPORT Investigators | JAMA | 1995 |
| 108 | Hanley, Lane, McBee, Ziai, Tuhrim, Lees, Dawson, Gandhi, Ullman, Mould, Mayo, Mendelow, Gregson | Thrombolytic removal of intraventricular haemorrhage in treatment of severe stroke: results of the randomised, multicentre, multiregion, placebo-controlled CLEAR III trial | Lancet | 2017 |
| 109 | Hanley, Thompson, Muschelli, Rosenblum, McBee, Lane, Bistran-Hall, Mayo, Keyl, Gandhi, Morgan, Ullman, Mould | Safety and efficacy of minimally invasive surgery plus alteplase in intracerebral haemorrhage evacuation (MISTIE): a randomised, controlled, open-label, phase 2 trial | Lancet Neurology | 2016 |
| 110 | Hanley, Thompson, Rosenblum, Yenokyan, Lane, McBee, Mayo, Bistran-Hall, Gandhi, Mould, Ullman, Ali, Carhuapoma, Kase, Lees, Dawson | Efficacy and safety of minimally invasive surgery with thrombolysis in intracerebral haemorrhage evacuation (MISTIE III): a randomised, controlled, open-label, blinded endpoint phase 3 trial | Lancet | 2019 |
| 111 | Hattori, Katayama, Maya, Gatherer | Impact of stereotactic hematoma evacuation on medical costs during the chronic period in patients with spontaneous putaminal hemorrhage: a randomized study | Surgical Neurology | 2006 |
| 112 | He, Zhang, Zhang, Jian, Deng, Yang, Xiao, Yu, Wen, Huang | Serum Procalcitonin Levels are Associated with Clinical Outcome in Intracerebral Hemorrhage | Cellular and Molecular Neurobiology | 2018 |
| 113 | Heard, Fink, Gamelli, Solomkin, Joshi, Trask, Fabian, Hudson, Gerold, Logan | Effect of prophylactic administration of recombinant human granulocyte colony-stimulating factor (filgrastim) on the frequency of nosocomial infections in patients with acute traumatic brain injury or cerebral hemorrhage | Critical Care Medicine | 1998 |
| 114 | Hegde and Menon | Modifying the Intracerebral Hemorrhage Score to Suit the Needs of the Developing World | Annals of the Indian Academy of Neurology | 2018 |
| 115 | Hemphill, Farrant, Neill | Prospective validation of the ICH Score for 12-month functional outcome | Neurology | 2009 |
| 116 | Honner, Singh, Cheung, Alter, Dutaret, Patel, Acharya | Emergency department control of blood pressure in intracerebral hemorrhage | The Journal of Emergency Medicine | 2011 |
| 117 | Hou, Liu, Zhang, Wang, Zheng | Evaluation of the Efficacy and Safety of Short-Course Deep Sedation Therapy for the Treatment of Intracerebral Hemorrhage After Surgery: A Non-Randomized Control Study | Medical Science Monitor | 2016 |
| 118 | Hsiang, Zhu, Wong, Kay, Poon | Putaminal and thalamic hemorrhage in ethnic chinese living in Hong Kong | Surgical Neurology | 1996 |
| 119 | Hu, Wang, Zhu, Yao, Chen, Xu, Lu | Circulating Klotho is linked to prognosis of acute intracerebral hemorrhage | Clinica Chimica Acta | 2019 |
| 120 | Huang and Zuo | RELEVANCE BETWEEN RANDOM BLOOD GLUCOSE LEVEL OF SPONTANEOUS CEREBRAL HEMORRHAGE IN ACUTE STAGE AND SEVERITY OF DISEASE AS WELL AS PROGNOSIS | Acta Medica Mediterranea | 2019 |
| 121 | Huang, Chen, Zhong, Yuan | Role of APACHE II scoring system in the prediction of severity and outcome of acute intracerebral hemorrhage | International Journal of Neuroscience | 2016 |
| 122 | Huang, Lo, Chang, Chen | Testing the outcome score of spontaneous intracerebral haemorrhage in haemodialysis patients | Internal Medicine Journal | 2009 |
| 123 | Hwang, Dell, Sparks, Watson, Langefeld, Comeau, Rosand, Battey, Koch | Clinician judgment vs formal scales for predicting intracerebral hemorrhage outcomes | Neurology | 2016 |
| 124 | Ibrahim, A. | Comparison between modified neuroendoscopy and craniotomy evacuation of spontaneous intra-cerebral hemorrhages: study of clinical outcome and glasgow outcome score | Bali Medical Journal | 2017 |
| 125 | Iemolo, Sanzaro, Duro, Giordano, Paciaroni | The prognostic value of biomarkers in stroke | Immunity and Ageing | 2016 |
| 126 | Imberti, Barillari, Biasioli, Bianchi, Contino, Duce, D'Inca, Mameli, Pinna, Ageno | Prothrombin complex concentrates for urgent anticoagulation reversal in patients with intracranial haemorrhage | Pathophysiology of Hemostasis and Thrombosis | 2008 |
| 127 | Imberti, Pietrobono, Klersy, Gamba, Iotti, Cornara | Intraoperative intravenous administration of rFVIIa and hematoma volume after early surgery for spontaneous intracerebral hemorrhage: A randomized prospective phase II study | Minerva Anestesiologica | 2012 |
| 128 | Inamasu, Nakae, Adachi, Hirose | Angiotensin II receptor blockers following intravenous nicardipine administration to lower blood pressure in patients with hypertensive intracerebral hemorrhage: a prospective randomized study | Blood Pressure Monitoring | 2017 |
| 129 | Investigators of RIGHT-2 | Prehospital transdermal glyceryl trinitrate in patients with ultra-acute presumed stroke (RIGHT-2): an ambulance-based, randomised, sham-controlled, blinded, phase 3 trial | Lancet | 2019 |
| 130 | Investigators of the ENOS trial | Efficacy of nitric oxide, with or without continuing antihypertensive treatment, for management of high blood pressure in acute stroke (ENOS): a partial-factorial randomised controlled trial | Lancet | 2015 |
| 131 | Jain, Jain, Bellolio, Schears, Rabinstein, Ganti | Is Early DNR a Self-Fulfilling Prophecy for Patients with Spontaneous Intracerebral Hemorrhage? | Neurocritical Care | 2013 |
| 132 | Ji, Chou, Wu, Shen, Yang, Wang, Lan, Shi | Association between serum periostin concentrations and outcome after acute spontaneous intracerebral hemorrhage | Clinica Chimica Acta | 2017 |
| 133 | Junttila, Ala-Kokko, Ohtonen, Vaarala, Karttunen, Vuolteenaho, Salo, Sutinen, Karhu, Herzig, Koskenkari | Neurogenic pulmonary edema in patients with nontraumatic intracerebral hemorrhage: predictors and association with outcome | Anesthesia and Analgesia | 2013 |
| 134 | Juvela | Risk factors for impaired outcome after spontaneous intracerebral hemorrhage | Archives of Neurology | 1995 |
| 135 | Juvela, Heiskanen, Poranen, Valtonen, Kuurne, Kaste, Troupp | The treatment of spontaneous intracerebral hemorrhage. A prospective randomized trial of surgical and conservative treatment | Journal of Neurosurgery | 1989 |
| 136 | Kalita, Bastia, Bhoi, Misra | Systemic Inflammatory Response Syndrome Predicts Severity of Stroke and Outcome | Journal of Stroke and Cerebrovascular Diseases | 2015 |
| 137 | Khodabandehlou, R.; Etemadyfar, M.; Nasr Esfahani, A. H. | Distribution by location of CT-diagnosed primary intracerebral hemorrhage in Isfahan | Journal of Research in Medical Sciences | 2005 |
| 138 | Kimura, Kazumi; Iguchi, Yasuyuki; Inoue, Takeshi; Shibazaki, Kensaku; Matsumoto, Noriko; Kobayashi, Kazuto; Yamashita, Shinji | Hyperglycemia independently increases the risk of early death in acute spontaneous intracerebral hemorrhage | Journal of the Neurological Sciences | 2007 |
| 139 | Kiyohara, Yutaka; Kubo, Michiaki; Kato, Isao; Tanizaki, Yimihiro; Tanaka, Keiichi; Okubo, Ken; Nakamura, Hidetoshi; Iida, Mitsuo | Ten-year prognosis of stroke and risk factors for death in a Japanese community: the Hisayama study | Stroke | 2003 |
| 140 | Knight, R. A.; Nagaraja, T. N.; Li, L.; Jiang, Q.; Tundo, K.; Chopp, M.; Seyfried, D. M. | A Prospective Safety Trial of Atorvastatin Treatment to Assess Rebleeding after Spontaneous Intracerebral Hemorrhage: A Serial MRI Investigation | Austin Journal of Cerebrovascular Diseases and Stroke | 2016 |
| 141 | Koga, Masatoshi; Toyoda, Kazunori; Yamagami, Hiroshi; Okuda, Satoshi; Okada, Yasushi; Kimura, Kazumi; Shiokawa, Yoshiaki; Nakagawara, Jyoji; Furui, Eisuke; Hasegawa, Yasuhiro; Kario, Kazuomi; Osaki, Masato; Miyagi, Tetsuya; Endo, Kaoru; Nagatsuka, Kazuyuki; Minematsu, Kazuo; Stroke Acute Management with Urgent Risk-factor, Assessment; Improvement Study, Investigators | Systolic blood pressure lowering to 160 mmHg or less using nicardipine in acute intracerebral hemorrhage: a prospective, multicenter, observational study (the Stroke Acute Management with Urgent Risk-factor Assessment and Improvement-Intracerebral Hemorrhage study) | Journal of Hypertension | 2012 |
| 142 | Kollmar, Rainer; Staykov, Dimitre; Dorfler, Arnd; Schellinger, Peter D.; Schwab, Stefan; Bardutzky, Jurgen | Hypothermia reduces perihemorrhagic edema after intracerebral hemorrhage | Stroke | 2010 |
| 143 | Kreel, L.; Kay, R.; Woo, J.; Wong, H. Y.; Nicholls, M. G. | THE RADIOLOGICAL (CT) AND CLINICAL SEQUELAE OF PRIMARY INTRACEREBRAL HEMORRHAGE | The British Journal of Radiology | 1991 |
| 144 | Kusano, Y.; Seguchi, T.; Horiuchi, T.; Kakizawa, Y.; Kobayashi, T.; Tanaka, Y.; Seguchi, K.; Hongo, K. | Prediction of functional outcome in acute cerebral hemorrhage using diffusion tensor imaging at 3T: a prospective study | American Journal of Neuroradiology | 2009 |
| 145 | Kuznietsov, A. A. | Development of multivariate models for the verification of short-term vital and functional prognosis in patients with hemorrhagic hemispheric stroke in the onset of the disease | Pathologia | 2018 |
| 146 | Kuzu, Yasutaka; Inoue, Takashi; Kanbara, Yoshiyuki; Nishimoto, Hideaki; Fujiwara, Shunro; Ogasawara, Kuniaki; Ogawa, Akira | Prediction of motor function outcome after intracerebral hemorrhage using fractional anisotropy calculated from diffusion tensor imaging | Cerebrovascular Diseases | 2012 |
| 147 | Kwan, J.; Hand, P. | Early neurological deterioration in acute stroke: clinical characteristics and impact on outcome | Quarterly Journal of Medicine | 2006 |
| 148 | Kwarisiima, L.; Mukisa, R.; Nakibuuka, J.; Matovu, S.; Katabira, E. | Thirty-day stroke mortality and associated clinical and laboratory factors among adult stroke patients admitted at Mulago Hospital (Uganda) | African Journal of Neurological Sciences | 2014 |
| 149 | Lacut K, Bressollette L. Le Gal G. Etienne E. De Tinteniac A. Renault A. Rouhart F. Besson G. Garcia J. F. Mottier D. Oger E. VICTORIAh Investigators | Prevention of venous thrombosis in patients with acute intracerebral hemorrhage | Neurology | 2005 |
| 150 | Lai, Yun-Ru; Lin, Yu-Jun; Shih, Fu-Yuan; Wang, Hung-Chen; Tsai, Nai-Wen; Kung, Chia-Te; Lin, Wei-Che; Cheng, Ben-Chung; Su, Yu-Jih; Chang, Ya-Ting; Su, Chih-Min; Hsiao, Sheng-Yuan; Huang, Chih-Cheng; Lu, Cheng-Hsien | Effect of Baroreceptor Sensitivity on Outcomes in Patients with Acute Spontaneous Intracerebral Hemorrhage | World Neurosurgery | 2018 |
| 151 | Landreneau, Margaret J.; Mullen, Michael T.; Messe, Steven R.; Cucchiara, Brett; Sheth, Kevin N.; McCullough, Louise D.; Kasner, Scott E.; Sansing, Lauren H.; Serum Markers After Spontaneous Cerebral Hemorrhage, Investigators | CCL2 and CXCL10 are associated with poor outcome after intracerebral hemorrhage | Annals of Clinical and Translational Neurology | 2018 |
| 152 | Lang, W. | Efficacy of Nitric Oxide, With or Without Continuing Antihypertensive Treatment, for Management of High Blood Pressure in Acute Stroke (ENOS): A Partial-Factorial Randomised Controlled Trial | Lancet | 2015 |
| 153 | Langhorne, P.; Wu, O.; Rodgers, H.; Ashburn, A.; Bernhardt, J. | A very early rehabilitation trial after stroke (AVERT): a Phase III, multicentre, randomised controlled trial | Health Technology Assessment | 2017 |
| 154 | Lauria, G.; Gentile, M.; Fassetta, G.; Casetta, I.; Agnoli, F.; Andreotta, G.; Barp, C.; Caneve, G.; Cavallaro, A.; Cielo, R. | Incidence and prognosis of stroke in the Belluno province, Italy. First-year results of a community-based study | Stroke | 1992 |
| 155 | Lauridsen, Signe Voigt; Hvas, Anne-Mette; Sandgaard, Emilie; Gyldenholm, Tua; Rahbek, Christian; Hjort, Niels; Tonnesen, Else Kirstine; Hvas, Christine Lodberg | Coagulation Profile after Spontaneous Intracerebral Hemorrhage: A Cohort Study | Journal of Stroke and Cerebrovascular Diseases | 2018 |
| 156 | Lee, H. K.; Ghani, A. R. I.; Awang, M. S.; Sayuthi, S.; Idris, B.; Abdullah, J. M. | Role of High Augmentation Index in Spontaneous Intracerebral Haemorrhage | Asian Journal of Surgery | 2010 |
| 157 | Lee, S. H.; Kim, B. J.; Ryu, W. S.; Kim, C. K.; Kim, N.; Park, B. J.; Yoon, B. W. | White matter lesions and poor outcome after intracerebral hemorrhage: a nationwide cohort study | Neurology | 2010 |
| 158 | Lees Kr, Sharma A. K. Barer D. Ford G. A. Kostulas V. Cheng Y. F. Odergren T. | Tolerability and pharmacokinetics of the nitrone NXY-059 in patients with acute stroke | Stroke | 2001 |
| 159 | Lei, C. Y.; Wu, B.; Liu, M.; Cao, T.; Wang, Q. X.; Dong, W. | Differences Between Vascular Structural Abnormality and Hypertensive Intracerebral Hemorrhage | Journal of Stroke and Cerebrovascular Diseases | 2015 |
| 160 | Leira, R.; Davalos, A.; Silva, Y.; Gil-Peralta, A.; Tejada, J.; Garcia, M.; Castillo, J.; Stroke Project, Cerebrovascular Diseases Group of the Spanish Neurological Society | Early neurologic deterioration in intracerebral hemorrhage: predictors and associated factors | Neurology | 2004 |
| 161 | Leno, C.; Berciano, J.; Combarros, O.; Polo, J. M.; Pascual, J.; Quintana, F.; Merino, J.; Sedano, C.; Martinduran, R.; Alvarez, C.; Llorca, J. | A PROSPECTIVE-STUDY OF STROKE IN YOUNG-ADULTS IN CANTABRIA, SPAIN | Stroke | 1993 |
| 162 | Li, Fei; Chen, Qian-Xue; Xiang, Shou-Gui; Yuan, Shi-Zhun; Xu, Xi-Zhen | The role of N-terminal pro-brain natriuretic peptide in evaluating the prognosis of patients with intracerebral hemorrhage | Journal of Neurology | 2017 |
| 163 | Li, Heng-Jie; Han, Nan-Nan; Nan, Yong; Zhang, Ke; Li, Gang; Chen, Huan | Plasma osteopontin acts as a prognostic marker in acute intracerebral hemorrhage patients | Clinica Chimica Acta | 2019 |
| 164 | Li, Jing-Ya; Yuan, Li-Xin; Zhang, Gen-Ming; Zhou, Li; Gao, Ying; Li, Qing-Bin; Chen, Che | Activating blood circulation to remove stasis treatment of hypertensive intracerebral hemorrhage: A multi-center prospective randomized open-label blinded-endpoint trial | Chinese Journal of Integrative Medicine | 2016 |
| 165 | Li, N.; Worthmann, H.; Heeren, M.; Schuppner, R.; Deb, M.; Tryc, A. B.; Bueltmann, E.; Lanfermann, H.; Donnerstag, F.; Weissenborn, K.; Raab, P. | Temporal pattern of cytotoxic edema in the perihematomal region after intracerebral hemorrhage: A serial magnetic resonance imaging study | Stroke | 2013 |
| 166 | Li, Na; Liu, Yan Fang; Ma, Li; Worthmann, Hans; Wang, Yi Long; Wang, Yong Jun; Gao, Yi Pei; Raab, Peter; Dengler, Reinhard; Weissenborn, Karin; Zhao, Xing Quan | Association of molecular markers with perihematomal edema and clinical outcome in intracerebral hemorrhage | Stroke | 2013 |
| 167 | Li, Na; Wang, Yilong; Wang, Wenjuan; Ma, Li; Xue, Jing; Weissenborn, Karin; Dengler, Reinhard; Worthmann, Hans; Wang, David Z.; Gao, Peiyi; Liu, Liping; Wang, Yongjun; Zhao, Xingquan | Contrast extravasation on computed tomography angiography predicts clinical outcome in primary intracerebral hemorrhage: a prospective study of 139 cases | Stroke | 2011 |
| 168 | Li, Q.; Yang, W. S.; Shen, Y. Q.; Xie, X. F.; Li, R.; Deng, L.; Yang, T. T.; Lv, F. J.; Lv, F. R.; Wu, G. F.; Tang, Z. P.; Goldstein, J. N.; Xie, P. | Benign Intracerebral Hemorrhage: A Population at Low Risk for Hematoma Growth and Poor Outcome | Journal of American Heart Association | 2019 |
| 169 | Li, Qi; Liu, Qing-Jun; Yang, Wen-Song; Wang, Xing-Chen; Zhao, Li-Bo; Xiong, Xin; Li, Rui; Cao, Du; Zhu, Dan; Wei, Xiao; Xie, Peng | Island Sign: An Imaging Predictor for Early Hematoma Expansion and Poor Outcome in Patients With Intracerebral Hemorrhage | Stroke | 2017 |
| 170 | Li, Weihua; Gao, Jianmei; Wei, Shufang; Wang, Donghai | Application values of clinical nursing pathway in patients with acute cerebral hemorrhage | Experimental and Therapeutic Medicine | 2016 |
| 171 | Li, X. W.; Li, J. Z.; Yang, X. H.; Sun, Z. S.; Zhang, J. R.; Zhao, W. M.; Dong, S. Z.; Li, C.; Ye, Y. Q.; Chen, J. C.; Li, Y. Q.; Xiang, Y.; Mao, J. H.; Li, G. J.; Guo, H.; Zhang, W. C.; Zhang, Y. Z.; Zhang, M. Z.; Zhang, W. Z.; Xu, Z. Y.; Zhao, B. S.; Wei, J. H.; Zhao, G. S.; Ma, R. H.; Shen, X. Z.; Ge, C. Y.; Zheng, C. L.; Li, S.; Wang, Y. | Hyperbaric-Oxygen Therapy Improves Survival and Functional Outcome of Acute Severe Intracerebral Hemorrhage | Archives of Medical Research | 2017 |
| 172 | Li, Xiaowei; Sun, Zhaosheng; Zhao, Wangmiao; Zhang, Jinrong; Chen, Jianchao; Li, Yongqian; Ye, Yanqiao; Zhao, Jinlian; Yang, Xuehui; Xiang, Yi; Li, Guangjie; Mao, Jianhui; Zhang, Wenchao; Zhang, Mingzhe; Zhang, Wanzeng | Effect of acetylsalicylic acid usage and platelet transfusion on postoperative hemorrhage and activities of daily living in patients with acute intracerebral hemorrhage | Journal of Neurosurgery | 2013 |
| 173 | Li, Zhong-Min; Zhang, Zhi-Ti; Guo, Chuan-Jun; Geng, Feng-Yang; Qiang, Fu; Wang, Le-Xin | Autologous bone marrow mononuclear cell implantation for intracerebral hemorrhage-a prospective clinical observation | Clinical Neurology and Neurosurgery | 2013 |
| 174 | Liao, H.; Xu, J.; Lin, Z. Z.; Yang, J. Y.; Chen, Q. A. | Effect of Tianhuang Granule (sic) on Intracranial Pressure and Serum Matrix Metalloproteinase-9 in Patients with Acute Cerebral Hemorrhage | Chinese Journal of Integrative Medicine | 2010 |
| 175 | Lima, T. T. F.; Prandini, M. N.; Gallo, P.; Cavalheiro, S. | Prognostic Value of Intraventricular Bleeding in Spontaneous Intraparenchymal Cerebral Hemorrhage of Small Volume: A Prospective Cohort Study | Neurosurgery | 2012 |
| 176 | Lin, X. F.; Ten, X. L.; Tang, X. B.; Chen, J. | Serum soluble CD40 ligand levels after acute intracerebral hemorrhage | Acta Neurologica Scandinavica | 2016 |
| 177 | Lindner, A.; Kofler, M.; Rass, V.; Ianosi, B.; Gaasch, M.; Schiefecker, A. J.; Beer, R.; Loveys, S.; Rhomberg, P.; Pfausler, B.; Thome, C.; Schmutzhard, E.; Helbok, R. | Early predictors for infectious complications in patients with spontaneous intracerebral hemorrhage and their impact on outcome | Frontiers in Neurology | 2019 |
| 178 | Liotta, Eric M.; Prabhakaran, Shyam; Sangha, Rajbeer S.; Bush, Robin A.; Long, Alan E.; Trevick, Stephen A.; Potts, Matthew B.; Jahromi, Babak S.; Kim, Minjee; Manno, Edward M.; Sorond, Farzaneh A.; Naidech, Andrew M.; Maas, Matthew B. | Magnesium, hemostasis, and outcomes in patients with intracerebral hemorrhage | Neurology | 2017 |
| 179 | Lioutas, V. A.; Goyal, N.; Katsanos, A. H.; Krogias, C.; Zand, R.; Sharma, V. K.; Varelas, P.; Malhotra, K.; Paciaroni, M.; Sharaf, A.; Chang, J.; Karapanayiotides, T.; Kargiotis, O.; Pappa, A.; Mai, J.; Pandhi, A.; Schroeder, C.; Tsantes, A.; Mehta, C.; Kerro, A.; Khan, A.; Mitsias, P. D.; Selim, M. H.; Alexandrov, A. V.; Tsivgoulis, G. | Clinical Outcomes and Neuroimaging Profiles in Nondisabled Patients With Anticoagulant-Related Intracerebral Hemorrhage | Stroke | 2018 |
| 180 | Liu, Bo-lin; Li, Bing; Zhang, Xiang; Fei, Zhou; Hu, Shi-jie; Lin, Wei; Gao, Da-kuan; Zhang, Li | A randomized controlled study comparing omeprazole and cimetidine for the prophylaxis of stress-related upper gastrointestinal bleeding in patients with intracerebral hemorrhage | Journal of Neurosurgery | 2013 |
| 181 | Liu, D. G.; Ding, H.; Liu, S. H.; Shen, J. Q. | Estimated glomerular filtration rate decline in 567 patients with acute stroke | Scandinavian Journal of Urology and Nephrology | 2012 |
| 182 | Liu, J.; Wang, D.; Yuan, R.; Xiong, Y.; Liu, M. | Prognosis of 908 patients with intracerebral hemorrhage in Chengdu, Southwest of China | International Journal of Neuroscience | 2017 |
| 183 | Liu, Ning; Cadilhac, Dominique A.; Andrew, Nadine E.; Zeng, Lingxia; Li, Zongfang; Li, Jin; Li, Yan; Yu, Xuewen; Mi, Baibing; Li, Zhe; Xu, Honghai; Chen, Yangjing; Wang, Juan; Yao, Wanxia; Li, Kuo; Yan, Feng; Wang, Jue | Randomized controlled trial of early rehabilitation after intracerebral hemorrhage stroke: difference in outcomes within 6 months of stroke | Stroke | 2014 |
| 184 | Lodder, J.; van Raak, L.; Hilton, A.; Hardy, E.; Kessels, A.; Group, Egasis Study | Diazepam to improve acute stroke outcome: results of the early GABA-Ergic activation study in stroke trial. a randomized double-blind placebo-controlled trial | Cerebrovascular Diseases | 2006 |
| 185 | Lorente, Leonardo; Martin, Maria M.; Abreu-Gonzalez, Pedro; Ramos, Luis; Argueso, Monica; Sole-Violan, Jordi; Caceres, Juan J.; Jimenez, Alejandro; Garcia-Marin, Victor | The Serum Melatonin Levels and Mortality of Patients with Spontaneous Intracerebral Hemorrhage | Brain Sciences | 2019 |
| 186 | Luan, Lei; Li, Maolei; Sui, Hang; Li, Guoliang; Pan, Wenyong | Efficacies of minimally invasive puncture and small bone window craniotomy for hypertensive intracerebral hemorrhage, evaluation of motor-evoked potentials and comparison of postoperative rehemorrhage between the two methods | Experimental and Therapeutic Medicine | 2019 |
| 187 | Luo Z-J, Guo T. M. Tu Q. Cheng X. L. Huang Y. Xiang M. Q. | Therapeutic effect of integrating Chinese patent medicine Xuesaitong Injection and western medicine in treating patients with hypertensive intracerebral hemorrhage: a prospective randomized controlled trial | European Journal of Integrative Medicine | 2018 |
| 188 | Luong, Chinh Quoc; Nguyen, Anh Dat; Nguyen, Chi Van; Mai, Ton Duy; Nguyen, Tuan Anh; Do, Son Ngoc; Dao, Phuong Viet; Pham, Hanh Thi My; Pham, Dung Thi; Ngo, Hung Manh; Nguyen, Quan Huu; Nguyen, Dat Tuan; Tran, Thong Huu; Le, Ky Van; Do, Nam Trong; Ngo, Ngoc Duc; Nguyen, Vinh Duc; Ngo, Hung Duc; Hoang, Hai Bui; Vu, Ha Viet; Vu, Lan Tuong; Ngo, Binh Thanh; Nguyen, Bai Xuan; Khuong, Dai Quoc; Nguyen, Dung Tien; Vuong, Trung Xuan; Be, Thu Hong; Gaberel, Thomas; Nguyen, Lieu Van | Effectiveness of Combined External Ventricular Drainage with Intraventricular Fibrinolysis for the Treatment of Intraventricular Haemorrhage with Acute Obstructive Hydrocephalus | Cerebrovascular Diseases | 2019 |
| 189 | Lyden Pd, Shuaib A. Lees K. R. Davalos A. Davis S. M. Diener H. C. Grotta J. C. Ashwood T. J. Hardemark H. G. Svensson H. H. Rodichok L. Wasiewski W. W. Ahlberg G. Chant Trial Investigators | Safety and tolerability of NXY-059 for acute intracerebral hemorrhage: the CHANT Trial | Stroke | 2007 |
| 190 | Lyden, P. D.; Shuaib, A.; Ng, K.; Atkinson, R.; Ashwood, T.; Nordlund, A.; Odergren, T. | The clomethiazole acute stroke study in hemorrhagic stroke (CLASS-H): Final results | Journal of Stroke and Cerebrovascular Diseases | 2000 |
| 191 | Ma, Chicheng; Liu, Aijun; Li, Zhenzuo; Zhou, Xueying; Zhou, Shengnian | Longitudinal study of diffusion tensor imaging properties of affected cortical spinal tracts in acute and chronic hemorrhagic stroke | Journal of Clinical Neuroscience | 2014 |
| 192 | Ma, Hongyin; Guo, Zhen-Ni; Liu, Jia; Xing, Yingqi; Zhao, Ren; Yang, Yi | Temporal Course of Dynamic Cerebral Autoregulation in Patients With Intracerebral Hemorrhage | Stroke | 2016 |
| 193 | Maas Mb, Berman M. D. Guth J. C. Liotta E. M. Prabhakaran S. Naidech A. M. | Neurochecks as a Biomarker of the Temporal Profile and Clinical Impact of Neurologic Changes after Intracerebral Hemorrhage | Journal of Stroke and Cerebrovascular diseases | 2015 |
| 194 | Maas, M. B.; Naidech, A. M.; Kim, M.; Batra, A.; Manno, E. M.; Sorond, F. A.; Prabhakaran, S.; Liotta, E. M. | Medication History versus Point-of-Care Platelet Activity Testing in Patients with Intracerebral Hemorrhage | Journal of Stroke and Cerebrovascular Diseases | 2018 |
| 195 | Maas, Matthew B.; Francis, Brandon A.; Sangha, Rajbeer S.; Lizza, Bryan D.; Liotta, Eric M.; Naidech, Andrew M. | Refining Prognosis for Intracerebral Hemorrhage by Early Reassessment | Cerebrovascular Diseases | 2017 |
| 196 | Maas, Matthew B.; Nemeth, Alexander J.; Rosenberg, Neil F.; Kosteva, Adam R.; Guth, James C.; Liotta, Eric M.; Prabhakaran, Shyam; Naidech, Andrew M. | Subarachnoid extension of primary intracerebral hemorrhage is associated with poor outcomes | Stroke | 2013 |
| 197 | Maas, Matthew B.; Nemeth, Alexander J.; Rosenberg, Neil F.; Kosteva, Adam R.; Prabhakaran, Shyam; Naidech, Andrew M | Delayed intraventricular hemorrhage is common and worsens outcomes in intracerebral hemorrhage | Neurology | 2013 |
| 198 | Maohong, C.; Kaifu, K.; Haihua, S. | Effects of prostaglandin E1 on perihematomal tissue after hypertensive intracerebral hemorrhage | Acta Neurologica Taiwanica | 2011 |
| 199 | Mapoure, Yacouba Njankouo; Ayeah, Chia Mark; Ba, Hamadou; Ngahane, Hugo Bertrand Mbatchou; Hentchoya, Romuald; Luma, Henry Namme | The prognostic value of serum uric acid in the acute phase of hemorrhagic stroke patients in black Africans | The Pan African Medical Journal | 2019 |
| 200 | Mapoure, Yacouba Njankouo; Eyambe, Ngowo Lydia; Dzudie, Anastase Tamdja; Ayeah, Chia Mark; Ba, Hamadou; Hentchoya, Romuald; Luma, Henry Namme | Gender-Related Differences and Short-Term Outcome of Stroke: Results from a Hospital-Based Registry in Sub-Saharan Africa | Neuroepidemiology | 2017 |
| 201 | Marquardt, G.; Wolff, R.; Janzen, R. W. C.; Seifert, V. | Basal ganglia haematomas in non-comatose patients: subacute stereotactic aspiration improves long-term outcome in comparison to purely medical treatment | Neurosurgical Review | 2005 |
| 202 | Marti-Fabregas, J.; Belvis, R.; Guardia, E.; Cocho, D.; Munoz, J.; Marruecos, L.; Marti-Vilalta, J. L. | Prognostic value of Pulsatility Index in acute intracerebral hemorrhage | Neurology | 2003 |
| 203 | Marti-Fabregas, J.; Delgado-Mederos, R.; Marin, R.; de la Ossa, N. P.; de Lecinana, M. A.; Rodriguez-Yanez, M.; Sanahuja, J.; Purroy, F.; De Arce, A. M.; Carrera, D.; Dinia, L.; Guardia-Laguarta, C.; Lleo, A. | Prognostic Value of Plasma beta-Amyloid Levels in Patients With Acute Intracerebral Hemorrhage | Stroke | 2014 |
| 204 | Mayda-Domac, F.; Misirli, H.; Yilmaz, M. | Prognostic Role of Mean Platelet Volume and Platelet Count in Ischemic and Hemorrhagic Stroke | Journal of Stroke and Cerebrovascular diseases | 2010 |
| 205 | Mayer Sa, Brun N. C. Broderick J. Davis S. Diringer M. N. Skolnick B. E. Steiner T. Europe AustralAsia NovoSeven I. C. H. Trial Investigators | Safety and feasibility of recombinant factor VIIa for acute intracerebral hemorrhage | Stroke; a journal of cerebral circulation | 2005 |
| 206 | Mayer, Stephan A.; Brun, Nikolai C.; Begtrup, Kamilla; Broderick, Joseph; Davis, Stephen; Diringer, Michael N.; Skolnick, Brett E.; Steiner, Thorsten; Investigators, Fast Trial | Efficacy and safety of recombinant activated factor VII for acute intracerebral hemorrhage | New England journal of medicine | 2008 |
| 207 | Mayer, Stephan A.; Brun, Nikolai C.; Begtrup, Kamilla; Broderick, Joseph; Davis, Stephen; Diringer, Michael N.; Skolnick, Brett E.; Steiner, Thorsten; Recombinant Activated Factor, V. I. I. Intracerebral Hemorrhage Trial Investigators | Recombinant activated factor VII for acute intracerebral hemorrhage | New England journal of medicine | 2005 |
| 208 | Mazaheri, Shahir; Reisi, Elahe; Poorolajal, Jalal; Ghiasian, Masoud | C-Reactive Protein Levels and Clinical Outcomes in Stroke Patients: A Prospective Cohort Study | Archives of Iranian medicine | 2018 |
| 209 | Mazdeh, Mehrdokht; Taher, Abbas; Torabian, Saadat; Seifirad, Soroush | Effects of Normobaric Hyperoxia in Severe Acute Stroke: a Randomized Controlled Clinical Trial Study | Acta medica Iranica | 2015 |
| 210 | Mendelow, A. D.; Gregson, B. A.; Fernandes, H. M.; Murray, G. D.; Teasdale, G. M.; Hope, D. T.; Karimi, A.; Shaw, M. D. M.; Barer, D. H.; Investigators, Stich | Early surgery versus initial conservative treatment in patients with spontaneous supratentorial intracerebral haematomas in the International Surgical Trial in Intracerebral Haemorrhage (STICH): a randomised trial | Lancet | 2005 |
| 211 | Miao, Z. L.; Jiang, L.; Xu, X.; Chen, K. L.; Lu, X. J. | Microsurgical treatment assisted by intraoperative ultrasound localization: A controlled trial in patients with hypertensive basal ganglia hemorrhage | British Journal of Neurosurgery | 2014 |
| 212 | Miller Cm, Vespa P. Saver J. L. Kidwell C. S. Carmichael S. T. Alger J. Frazee J. Starkman S. Liebeskind D. Nenov V. Elashoff R. Martin N. | Image-guided endoscopic evacuation of spontaneous intracerebral hemorrhage | Surgical Neurology | 2008 |
| 213 | Misra, U. K.; Kalita, J.; Pandey, S.; Mandal, S. K. | Predictors of gastrointestinal bleeding in acute intracerebral haemorrhage | Journal of the neurological Sciences | 2003 |
| 214 | Misra, U. K.; Kalita, J.; Pandey, S.; Mandal, S. K.; Srivastava, M | A randomized placebo controlled trial of ranitidine versus sucralfate in patients with spontaneous intracerebral hemorrhage for prevention of gastric hemorrhage | Journal of the neurological Sciences | 2005 |
| 215 | Misra, U. K.; Kalita, J.; Ranjan, P.; Mandal, S. K. | Mannitol in intracerebral hemorrhage: a randomized controlled study | Journal of the neurological Sciences | 2005 |
| 216 | Miyagi, Tetsuya; Koga, Masatoshi; Yamagami, Hiroshi; Okuda, Satoshi; Okada, Yasushi; Kimura, Kazumi; Shiokawa, Yoshiaki; Nakagawara, Jyoji; Furui, Eisuke; Hasegawa, Yasuhiro; Kario, Kazuomi; Arihiro, Shoji; Sato, Shoichiro; Minematsu, Kazuo; Toyoda, Kazunori | Reduced estimated glomerular filtration rate affects outcomes 3 months after intracerebral hemorrhage: the stroke acute management with urgent risk-factor assessment and improvement-intracerebral hemorrhage study | Journal of stroke and cerebrovascular diseases : the official journal of National Stroke Association | 2015 |
| 217 | Morgenstern Lb, Frankowski R. F. Shedden P. Pasteur W. Grotta J. C. | Surgical treatment for intracerebral hemorrhage (STICH): a single-center, randomized clinical trial | Neurology | 1998 |
| 218 | Morgenstern, L. B.; Demchuk, A. M.; Kim, D. H.; Frankowski, R. F.; Grotta, J. C. | Rebleeding leads to poor outcome in ultra-early craniotomy for intracerebral hemorrhage | Neurology | 2001 |
| 219 | Morgenstern, Lewis B.; Zahuranec, Darin B.; Sanchez, Brisa N.; Becker, Kyra J.; Geraghty, Madeleine; Hughes, Rebecca; Norris, Gregory; Hemphill, J. Claude, 3rd | Full medical support for intracerebral hemorrhage | Neurology | 2015 |
| 220 | Moulin, T.; Tatu, L.; Crepin-Leblond, T.; Chavot, D.; Berges, S.; Rumbach, T. | The Besancon Stroke Registry: an acute stroke registry of 2,500 consecutive patients | European Neurology | 1997 |
| 221 | Mourad Hs, Enab A. A. Abdelalim A. M. | Early outcome of conservative versus surgical treatment of Spontaneous supratentorial intracerebral hemorrhage | Egyptian Journal of Neurology, Psychiatry, and Neurosurgery | 2011 |
| 222 | Murthy, Santosh B.; Levy, Andrew P.; Duckworth, Joshua; Schneider, Eric B.; Shalom, Hadar; Hanley, Daniel F.; Tamargo, Rafael J.; Nyquist, Paul A. | Presence of haptoglobin-2 allele is associated with worse functional outcomes after spontaneous intracerebral hemorrhage | World neurosurgery | 2015 |
| 223 | Nag Dr, C.; Das, K.; Ghosh, M.; Khandakar, M. R. | Plasma osmolality in acute spontanious intra-cerebral hemorrhage: Does it influence hematoma volume and clinical outcome? | Journal of Research in Medical Sciences | 2012 |
| 224 | Nag, C.; Das, K.; Ghosh, M.; Khandakar, M. R. | Prediction of clinical outcome in acute hemorrhagic stroke from a single CT scan on admission | North American Journal of Medical Sciences | 2012 |
| 225 | Naidech, A. M.; Beaumont, J. L.; Berman, M.; Francis, B.; Liotta, E.; Maas, M. B.; Prabhakaran, S.; Holl, J.; Cella, D. | Dichotomous good outcome indicates mobility more than cognitive or social quality of life | Critical Care Medicine | 2015 |
| 226 | Naidech, A. M.; Liebling, S. M.; Rosenberg, N. F.; Lindholm, P. F.; Bernstein, R. A.; Batjer, H. H.; Alberts, M. J.; Kwaan, H. C. | Early Platelet Transfusion Improves Platelet Activity and May Improve Outcomes After Intracerebral Hemorrhage | Neurocritical Care | 2012 |
| 227 | Naidech, A. M.; Rosenberg, N. F.; Bernstein, R. A.; Batjer, H. H. | Aspirin use or reduced platelet activity predicts craniotomy after intracerebral hemorrhage | Neurocritical Care | 2011 |
| 228 | Naidech, Andrew M.; Beaumont, Jennifer L.; Rosenberg, Neil F.; Maas, Matthew B.; Kosteva, Adam R.; Ault, Michael L.; Cella, David; Ely, E. Wesley | Intracerebral hemorrhage and delirium symptoms. Length of stay, function, and quality of life in a 114-patient cohort | American journal of respiratory and critical care medicine | 2013 |
| 229 | Naidech, Andrew M.; Beaumont, Jennifer; Muldoon, Kathryn; Liotta, Eric M.; Maas, Matthew B.; Potts, Matthew B.; Jahromi, Babak S.; Cella, David; Prabhakaran, Shyam; Holl, Jane L. | Prophylactic Seizure Medication and Health-Related Quality of Life After Intracerebral Hemorrhage | Critical Care Medicine | 2018 |
| 230 | Naidech, Andrew M.; Bendok, Bernard R.; Garg, Rajeev K.; Bernstein, Richard A.; Alberts, Mark J.; Bleck, Thomas P.; Batjer, H. Hunt | Reduced platelet activity is associated with more intraventricular hemorrhage | Neurosurgery | 2009 |
| 231 | Naidech, Andrew M.; Bernstein, Richard A.; Bassin, Sarice L.; Garg, Rajeev K.; Liebling, Storm; Bendok, Bernard R.; Batjer, H. Hunt; Bleck, Thomas P. | How patients die after intracerebral hemorrhage | Neurocritical Care | 2009 |
| 232 | Naidech, Andrew M.; Bernstein, Richard A.; Levasseur, Kimberly; Bassin, Sarice L.; Bendok, Bernard R.; Batjer, H. Hunt; Bleck, Thomas P.; Alberts, Mark J. | Platelet activity and outcome after intracerebral hemorrhage | Annals of Neurology | 2009 |
| 233 | Naidech, Andrew M.; Garg, Rajeev K.; Liebling, Storm; Levasseur, Kimberly; Macken, Micheal P.; Schuele, Stephan U.; Batjer, H. Hunt | Anticonvulsant use and outcomes after intracerebral hemorrhage | Stroke | 2009 |
| 234 | Naidech, Andrew M.; Jovanovic, Borko; Liebling, Storm; Garg, Rajeev K.; Bassin, Sarice L.; Bendok, Bernard R.; Bernstein, Richard A.; Alberts, Mark J.; Batjer, H. Hunt | Reduced platelet activity is associated with early clot growth and worse 3-month outcome after intracerebral hemorrhage | Stroke | 2009 |
| 235 | Nakibuuka, Jane; Sajatovic, Martha; Nankabirwa, Joaniter; Ssendikadiwa, Charles; Furlan, Anthony J.; Katabira, Elly; Kayima, James; Kalema, Nelson; Byakika-Tusiime, Jayne; Ddumba, Edward | Early mortality and functional outcome after acute stroke in Uganda: prospective study with 30 day follow-up | Springer Plus | 2015 |
| 236 | Newell, D. W.; Shah, M. M.; Wilcox, R.; Hansmann, D. R.; Melnychuk, E.; Muschelli, J.; Hanley, D. F. | Minimally invasive evacuation of spontaneous intracerebral hemorrhage using sonothrombolysis Clinical article | Journal of Neurosurgery | 2011 |
| 237 | O.P.M. Teernstra, MD; S.M.A.A. Evers, PhD; J. Lodder, MD; P. Leffers, MSc; C.L. Franke, MD; G. Blaauw, MD | Stereotactic treatment of intracerebral hematoma by means of a plasminogen activator: a multicenter randomized controlled trial (SICHPA) | Stroke | 2003 |
| 238 | Obiako, O. R.; Oparah, S. K.; Ogunniyi, A. | Prognosis and outcome of acute stroke in the University College Hospital Ibadan, Nigeria | Nigerian Journal of Clinical Practice | 2011 |
| 239 | Oertel, J. M. K.; Mondorf, Y.; BalDauf, J.; Schroeder, H. W. S.; Gaab, M. R. | Endoscopic third ventriculostomy for obstructive hydrocephalus due to intracranial hemorrhage with intraventricular extension Clinical article | Journal of Neurosurgery | 2009 |
| 240 | Ojha, Piyush; Sardana, Vijay; Maheshwari, Dilip; Bhushan, Bharat; Kamble, Sumit | Clinical Profile of Patients with Acute Intracerebral Hemorrhage and ICH Score as an Outcome Predictor on Discharge, 30 Days and 60 Days Follow-up | The Journal of the Association of Physicians of India | 2019 |
| 241 | Olowoyo, P.; Owolabi, M. O.; Fawale, B.; Ogunniyi, A. | Short term stroke outcome is worse among indiv1iduals with sickle cell trait | eNeurologicalSci | 2016 |
| 242 | Ong, T. Z.; Raymond, A. A. | Risk factors for stroke and predictors of one-month mortality | Singapore medical journal | 2002 |
| 243 | Osaki, M.; Koga, M.; Maeda, K.; Hasegawa, Y.; Nakagawara, J.; Furui, E.; Todo, K.; Kimura, K.; Shiokawa, Y.; Okada, Y.; Okuda, S.; Kario, K.; Yamagami, H.; Minematsu, K.; Kitazono, T.; Toyoda, K.; Stroke Acute Management, Urgent | A multicenter, prospective, observational study of warfarin-associated intracerebral hemorrhage: The SAMURAI-WAICH study | Journal of the Neurological Sciences | 2015 |
| 244 | Paciaroni, Maurizio; Mazzotta, Giovanni; Corea, Francesco; Caso, Valeria; Venti, Michele; Milia, Paolo; Silvestrelli, Giorgio; Palmerini, Francesco; Parnetti, Lucilla; Gallai, Virgilio | Dysphagia following Stroke | European Neurology | 2004 |
| 245 | Pan, W. H.; Lai, Y. H.; Yeh, W. T.; Chen, J. R.; Jeng, J. S.; Bai, C. H.; Lin, R. T.; Lee, T. H.; Chang, K. C.; Lin, H. J.; Hsiao, C. F.; Chern, C. M.; Lien, L. M.; Liu, C. H.; Chen, W. H.; Chang, A. | Intake of potassium- and magnesium-enriched salt improves functional outcome after stroke: A randomized, multicenter, double-blind controlled trial | American Journal of Clinical Nutrition | 2017 |
| 246 | Pantazis, Georgios; Tsitsopoulos, Parmenion; Mihas, Constantinos; Katsiva, Vasiliki; Stavrianos, Vasilios; Zymaris, Stylianos | Early surgical treatment vs conservative management for spontaneous supratentorial intracerebral hematomas: A prospective randomized study | Surgical Neurology | 2006 |
| 247 | Park, S. Y.; Kong, M. H.; Kim, J. H.; Kang, D. S.; Song, K. Y.; Huh, S. K. | Role of 'Spot sign' on ct angiography to predict hematoma expansion in spontaneous intracerebral hemorrhage | Journal of Korean Neurosurgical Society | 2010 |
| 248 | Patil, A. S.; Tikait, N. U.; Dange, N. N.; Chougule, S.; Patil, S. A. | COMPARISON OF CLINICAL PRESENTATION AND RISK FACTORS OF STROKE IN DIABETIC VERSUS NON-DIABETIC POPULATION-RESULTS OF A SINGLE CENTRE STUDY | Journal of Evolution of Medical and Dental Sciences - JEMDS | 2017 |
| 249 | Perez de la Ossa, Natalia; Sobrino, Tomas; Silva, Yolanda; Blanco, Miguel; Millan, Monica; Gomis, Meritxell; Agulla, Jesus; Araya, Pablo; Reverte, Silvia; Serena, Joaquin; Davalos, Antoni | Iron-related brain damage in patients with intracerebral hemorrhage | Stroke | 2010 |
| 250 | Perini, F.; Galloni, E.; Bolgan, I.; Bader, G.; Ruffini, R.; Arzenton, E.; Alba, S.; Azzini, C.; Bartolomei, L.; Billo, G.; Bortolon, F.; Dudine, P.; Garofalo, P. G.; L'Erario, R.; Morra, M.; Parisen, P.; Stenta, G.; Toso, V. | Elevated plasma homocysteine in acute stroke was not associated with severity and outcome: stronger association with small artery disease | Neurological Sciences | 2005 |
| 251 | Pias-Peleteiro, Juan; Perez-Mato, Maria; Lopez-Arias, Esteban; Rodriguez-Yanez, Manuel; Blanco, Miguel; Campos, Francisco; Castillo, Jose; Sobrino, Tomas | Increased Endothelial Progenitor Cell Levels are Associated with Good Outcome in Intracerebral Hemorrhage | Scientific Reports | 2016 |
| 252 | Pikija, Slaven; Cvetko, Danijel; Malojcic, Branko; Trkanjec, Zlatko; Pavlicek, Ivan; Lukic, Anita; Kopjar, Andrina; Hajduk, Martina; Androvic, Alen; Bilic-Genter, Melita; Trkulja, Vladimir | A population-based prospective 24-month study of stroke: incidence and 30-day case-fatality rates of first-ever strokes in Croatia | Neuroepidemiology | 2012 |
| 253 | Planton, M.; Saint-Aubert, L.; Raposo, N.; Branchu, L.; Lyoubi, A.; Bonneville, F.; Albucher, J. F.; Olivot, J. M.; Peran, P.; Pariente, J. | High prevalence of cognitive impairment after intracerebral hemorrhage | PLoS One | 2017 |
| 254 | Potter, John F.; Robinson, Thompson G.; Ford, Gary A.; Mistri, Amit; James, Martin; Chernova, Julia; Jagger, Carol | Controlling hypertension and hypotension immediately post-stroke (CHHIPS): a randomised, placebo-controlled, double-blind pilot trial | The Lancet Neurology | 2009 |
| 255 | Prodan, Calin I.; Stoner, Julie A.; Dale, George L. | Lower Coated-Platelet Levels Are Associated With Increased Mortality After Spontaneous Intracerebral Hemorrhage | Stroke | 2015 |
| 256 | Puig, J.; Blasco, G.; Terceno, M.; Daunis, I. Estadella P.; Schlaug, G.; Hernandez-Perez, M.; Cuba, V.; Carbo, G.; Serena, J.; Essig, M.; Figley, C. R.; Nael, K.; Leiva-Salinas, C.; Pedraza, S.; Silva, Y. | Predicting motor outcome in acute intracerebral hemorrhage | American Journal of Neuroradiology | 2019 |
| 257 | Qian, Song-Quan; He, Su-Rong; Li, Bei-Bei; Qian, Jing; Zheng, Xu-Dong | Serum S100A12 and 30-day mortality after acute intracerebral hemorrhage | International Journal of Clinical Chemistry | 2018 |
| 258 | Qian, Song-Quan; Hu, Xiao-Chun; He, Su-Rong; Li, Bei-Bei; Zheng, Xu-Dong; Pan, Guang-Hua | Prognostic value of serum thioredoxin concentrations after intracerebral hemorrhage | International Journal of Clinical Chemistry | 2016 |
| 259 | Qiu, Shen-Zhong; Wang, Hong-Xiang; Shen, Jia; Zheng, Guan-Rong; Chen, Bin; Huang, Jian-Jun; Gao, Jian-Bo | The prognostic value of serum signal peptide-Cub-Egf domain-containing protein-1 concentrations in acute intracerebral hemorrhage | International Journal of Clinical Chemistry | 2016 |
| 260 | Qureshi, A. I.; Palesch, Y. Y.; Barsan, W. G.; Hanley, D. F.; Hsu, C. Y.; Martin, R. L.; Moy, C. S.; Silbergleit, R.; Steiner, T.; Suarez, J. I.; Toyoda, K.; Wang, Y. J.; Yamamoto, H.; Yoon, B. W.; Investigators, Atach- Trial; Neurological Emergency Treatment, T. | Intensive Blood-Pressure Lowering in Patients with Acute Cerebral Hemorrhage | New England journal of medicine | 2016 |
| 261 | Qureshi, Adnan I.; Harris-Lane, Pansy; Kirmani, Jawad F.; Ahmed, Shafiuddin; Jacob, Molly; Zada, Yasin; Divani, Afshin A. | Treatment of acute hypertension in patients with intracerebral hemorrhage using American Heart Association guidelines | Critical Care Medicine | 2006 |
| 262 | Qureshi, Adnan I.; Mohammad, Yousef M.; Yahia, Abutaher M.; Suarez, Jose I.; Siddiqui, Amir M.; Kirmani, Jawad F.; Suri, M. Fareed K.; Kolb, James; Zaidat, Osama O. | A prospective multicenter study to evaluate the feasibility and safety of aggressive antihypertensive treatment in patients with acute intracerebral hemorrhage | Journal of Intensive Care Medicine | 2006 |
| 263 | Rahmani, F.; Rikhtegar, R.; Ala, A.; Farkhad-Rasooli, A.; Ebrahimi-Bakhtavar, H. | Predicting 30-day mortality in patients with primary intracerebral hemorrhage (ICH): Evaluation of the value of intracerebral hemorrhage and modified new intracerebral hemorrhage scores | Iranian Journal of Neurology | 2018 |
| 264 | Rainer, Timothy H.; Wong, Ka Sing; Lam, Wynnie; Lam, Nicole Y. L.; Graham, Colin A.; Lo, Y. M. Dennis | Comparison of plasma beta-globin DNA and S-100 protein concentrations in acute stroke | International Journal of Clinical Chemistry | 2007 |
| 265 | Rajapathy, S. K.; Idris, Z.; Kandasamy, R.; Wong, S. H. A.; Abdullah, J. M. | Inflammatory Biomarkers and Their Value in Predicting Survival and Outcome among Patients with Spontaneous Intracerebral Haemorrhage | Malaysian Journal of Medical Sciences | 2017 |
| 266 | Ramirez-Moreno, Jose Maria; Casado-Naranjo, Ignacio; Portilla, Juan Carlos; Calle, Maria Luisa; Tena, David; Falcon, Alfonso; Serrano, Ana | Serum cholesterol LDL and 90-day mortality in patients with intracerebral hemorrhage | Stroke | 2009 |
| 267 | Rashid, H. U.; Amin, R.; Rahman, A.; Islam, M. R.; Hossain, M.; Barua, K. K.; Hossain, M. A. | Correlation between intracerebral hemorrhage score and surgical outcome of spontaneous intracerebral hemorrhage | Bangladesh Medical Research Council bulletin | 2013 |
| 268 | Rasras, S.; Safari, H.; Zeinali, M.; Jahangiri, M. | Decompressive hemicraniectomy without clot evacuation in supratentorial deep-seated intracerebral hemorrhage | Clinical neurology and neurosurgery | 2018 |
| 269 | Rathor, Mohammad Yousuf; Rani, Mohammad Fauzi Abdul; Jamalludin, A. R.; Amran, M.; Shahrin, T. C. A.; Shah, A. | Prediction of functional outcome in patients with primary intracerebral hemorrhage by clinical-computed tomographic correlations | Journal of research in medical sciences : the official journal of Isfahan University of Medical Sciences | 2012 |
| 270 | Regenhardt, Robert W.; Biseko, Maijo R.; Shayo, Agness F.; Mmbando, Theoflo N.; Grundy, Sara J.; Xu, Ai; Saadi, Altaf; Wibecan, Leah; Kharal, G. Abbas; Parker, Robert; Klein, Joshua P.; Mateen, Farrah J.; Okeng'o, Kigocha | Opportunities for intervention: stroke treatments, disability and mortality in urban Tanzania | International journal for quality in health care : journal of the International Society for Quality in Health Care | 2019 |
| 271 | Richard, S.; Lagerstedt, L.; Burkhard, P. R.; Debouverie, M.; Turck, N.; Sanchez, J. C. | E-selectin and vascular cell adhesion molecule-1 as biomarkers of 3-month outcome in cerebrovascular diseases | Journal of Inflammation | 2015 |
| 272 | Rizos, Timolaos; Dorner, Nils; Jenetzky, Ekkehart; Sykora, Marek; Mundiyanapurath, Sibu; Horstmann, Solveig; Veltkamp, Roland; Rohde, Stefan; Bendszus, Martin; Steiner, Thorsten | Spot signs in intracerebral hemorrhage: useful for identifying patients at risk for hematoma enlargement? | Cerebrovascular Diseases | 2013 |
| 273 | Robinson, T. G.; Potter, J. F.; Ford, G. A.; Bulpitt, C.; Chernova, J.; Jagger, C.; James, M. A.; Knight, J.; Markus, H. S.; Mistri, A. K.; Poulter, N. R.; Investigators, Cossacs | Effects of antihypertensive treatment after acute stroke in the Continue Or Stop post-Stroke Antihypertensives Collaborative Study (COSSACS): a prospective, randomised, open, blinded-endpoint trial | Lancet Neurology | 2010 |
| 274 | Rodriguez-Fernandez, S.; Castillo-Lorente, E.; Guerrero-Lopez, F.; Rodriguez-Rubio, D.; Aguilar-Alonso, E.; Lafuente-Baraza, J.; Gomez-Jimenez, F. J.; Mora-Ordonez, J.; Rivera-Lopez, R.; Arias-Verdu, M. D.; Quesada-Garcia, G.; Arraez-Sanchez, M. A.; Rivera-Fernandez, R. | Validation of the ICH score in patients with spontaneous intracerebral haemorrhage admitted to the intensive care unit in Southern Spain | BMJ Open | 2018 |
| 275 | Rodriguez-Luna, D.; Pineiro, S.; Ribo, M.; Ibarra, B.; Rubiera, M.; Pagola, J.; Maisterra, O.; Coscojuela, P.; Romero, F.; Alvarez-Sabin, J.; Molina, C. A. | Ultraearly hematoma growth predicts poor outcome after acute intracerebral hemorrhage | Neurology | 2011 |
| 276 | Rodriguez-Luna, D.; Pineiro, S.; Ribo, M.; Ibarra, B.; Rubiera, M.; Pagola, J.; Maisterra, O.; Coscojuela, P.; Romero, F.; Alvarez-Sabin, J.; Molina, C. A. | Serum LDL cholesterol level predicts hematoma growth after acute intracerebral hemorrhage | Stroke | 2011 |
| 277 | Rodriguez-Yanez, Manuel; Brea, David; Arias, Susana; Blanco, Miguel; Pumar, Jose M.; Castillo, Jose; Sobrino, Tomas | Increased expression of Toll-like receptors 2 and 4 is associated with poor outcome in intracerebral hemorrhage | Journal of Neuroimmunology | 2012 |
| 278 | Ronning Om, Guldvog B. | Stroke units versus general medical wards, I: twelve- and eighteen-month survival: a randomized, controlled trial | Stroke; a journal of cerebral circulation | 1998 |
| 279 | Ronning Om, Guldvog B. Stavem K. | The benefit of an acute stroke unit in patients with intracranial haemorrhage: a controlled trial | Journal of neurology, neurosurgery, and psychiatry | 2001 |
| 280 | Ronning, O. M.; Stavem, K. | Predictors of mortality following acute stroke: A cohort study with 12 years of follow-up | Journal of Stroke and Cerebrovascular diseases | 2012 |
| 281 | Roquer, Jaume; Rodriguez Campello, Ana; Gomis, Meritxell; Ois, Angel; Puente, Victor; Munteis, Elvira | Previous antiplatelet therapy is an independent predictor of 30-day mortality after spontaneous supratentorial intracerebral hemorrhage | Journal of Neurology | 2005 |
| 282 | Rosand, J.; Eckman, M. H.; Knudsen, K. A.; Singer, D. E.; Greenberg, S. M | The effect of warfarin and intensity of anticoagulation on outcome of intracerebral Hemorrhage | Archives of Internal Medicine | 2004 |
| 283 | Rosenthal, Lisa J.; Francis, Brandon A.; Beaumont, Jennifer L.; Cella, David; Berman, Michael D.; Maas, Matthew B.; Liotta, Eric M.; Askew, Robert; Naidech, Andrew M. | Agitation, Delirium, and Cognitive Outcomes in Intracerebral Hemorrhage | Psychosomatics | 2017 |
| 284 | Sandset, Else Charlotte; Bath, Philip M. W.; Boysen, Gudrun; Jatuzis, Dalius; Korv, Janika; Luders, Stephan; Murray, Gordon D.; Richter, Przemyslaw S.; Roine, Risto O.; Terent, Andreas; Thijs, Vincent; Berge, Eivind; Group, Scast Study | The angiotensin-receptor blocker candesartan for treatment of acute stroke (SCAST): a randomised, placebo-controlled, double-blind trial | Lancet (London, England) | 2011 |
| 285 | Saver Jl, Starkman S. Eckstein M. Stratton S. J. Pratt F. D. Hamilton S. Conwit R. Liebeskind D. S. Sung G. Kramer I. Moreau G. Goldweber R. Sanossian N. Fast- M. A. G. Investigators; Coordinators | Prehospital use of magnesium sulfate as neuroprotection in acute stroke | New England journal of medicine | 2015 |
| 286 | Schellinger, Peter D.; Fiebach, Jochen B.; Hoffmann, Katrin; Becker, Kristina; Orakcioglu, Berk; Kollmar, Rainer; Juttler, Eric; Schramm, Peter; Schwab, Stefan; Sartor, Klaus; Hacke, Werner | Stroke MRI in intracerebral hemorrhage: is there a perihemorrhagic penumbra? | Stroke | 2003 |
| 287 | Schneider, D.; Berrouschot, J.; Brandt, T.; Hacke, W.; Ferbert, A.; Norris, S. H.; Polmar, S. H.; Schafer, E. | Safety, pharmacokinetics and biological activity of enlimomab (anti-ICAM-1 antibody): an open-label, dose escalation study in patients hospitalized for acute stroke | European Neurology | 1998 |
| 288 | Schneider, H.; Hertel, F.; Kuhn, M.; Ragaller, M.; Gottschlich, B.; Trabitzsch, A.; Dengl, M.; Neudert, M.; Reichmann, H.; Wopking, S. | Decannulation and Functional Outcome After Tracheostomy in Patients with Severe Stroke (DECAST): A Prospective Observational Study | Neurocritical Care | 2017 |
| 289 | Secades Jj, Alvarez-Sabin J. Rubio F. Lozano R. Davalos A. Castillo J. Trial Investigators | Citicoline in intracerebral haemorrhage: a double-blind, randomized, placebo-controlled, multi-centre pilot study | Cerebrovascular Diseases | 2006 |
| 290 | Selim, Magdy; Foster, Lydia D.; Moy, Claudia S.; Xi, Guohua; Hill, Michael D.; Morgenstern, Lewis B.; Greenberg, Steven M.; James, Michael L.; Singh, Vineeta; Clark, Wayne M.; Norton, Casey; Palesch, Yuko Y.; Yeatts, Sharon D.; i, D. E. F. Investigators | Deferoxamine mesylate in patients with intracerebral haemorrhage (i-DEF): a multicentre, randomised, placebo-controlled, double-blind phase 2 trial | Lancet Neurology | 2019 |
| 291 | Sembill, J. A.; Wieser, C. Y.; Sprugel, M. I.; Gerner, S. T.; Giede-Jeppe, A.; Reindl, C.; Eyupoglu, I. Y.; Hoelter, P.; Lucking, H.; Kuramatsu, J. B.; Huttner, H. B. | Initiating anticoagulant therapy after ICH is associated with patient characteristics and treatment recommendations | Journal of Neurology | 2018 |
| 292 | Seo, W.; Oh, H. | Comparisons of acute physiological parameters influencing outcome in patients with traumatic brain injury and hemorrhagic stroke | Worldviews on Evidence-Based Nursing | 2009 |
| 293 | Seo, WhaSook; Oh, HyunSoo | Acute physiologic predictors of mortality and functional and cognitive recovery in hemorrhagic stroke: 1-, 3-, and 6-month assessments | Journal of stroke and cerebrovascular diseases : the official journal of National Stroke Association | 2007 |
| 294 | Sharafadinzadeh N, Baghebanian S. M. Pipelzadeh M. Moravej Ale Ali A. Ghanavati P. | Effects of dexamethasone in primary intracerebral hemorrhage in the South West of Iran | Pakistan Journal of Medical Sciences | 2008 |
| 295 | Sharma, J. C.; Ananda, K.; Ross, I.; Hill, R.; Vassallo, M. | N-terminal proBrain Natriuretic Peptide Levels Predict Short-term Poststroke Survival | Journal of Stroke and Cerebrovascular diseases | 2006 |
| 296 | Shen, Q.; Cordato, D.; Chan, D. K. Y.; Hung, W. T.; Karr, M. | Identifying the determinants of 1-year post-stroke outcomes in elderly patients | Acta neurologica Scandinavica | 2006 |
| 297 | Sheng, Ai Zhen; Shen, Qing; Cordato, Dennis; Zhang, Yun Yun; Yin Chan, Daniel Kam | Delirium within three days of stroke in a cohort of elderly patients | Journal of the American Geriatrics Society | 2006 |
| 298 | Sibon, Igor; Lassalle-Lagadec, Saioa; Renou, Pauline; Swendsen, Joel | Evolution of depression symptoms following stroke: a prospective study using computerized ambulatory monitoring | Cerebrovascular Diseases | 2012 |
| 299 | Silva, Yolanda; Puigdemont, Montserrat; Castellanos, Mar; Serena, Joaquin; Suner, Rosa M.; Garcia, Maria M.; Davalos, Antoni | Semi-intensive monitoring in acute stroke and long-term outcome | Cerebrovascular Diseases | 2005 |
| 300 | Singh, A. V.; Singh, H. V.; Singh, S. | STUDY OF RENAL FUNCTION TESTS IN PATIENTS OF ACUTE HAEMORRHAGIC STROKE | Journal of Evolution of Medical and Dental Sciences | 2015 |
| 301 | Singh, Jittendra K.; Ranjan, Piyush; Kumari, Archana; Dahale, Amol S.; Jha, Rajendra; Das, Ranjan | Types, outcome and risk factors of stroke in Tribal Patients | International Journal of stroke | 2013 |
| 302 | Sobrino, T.; Arias, S.; Perez-Mato, M.; Agulla, J.; Brea, D.; Rodriguez-Yanez, M.; Castillo, J. | Cd34+progenitor cells likely are involved in the good functional recovery after intracerebral hemorrhage in humans | Journal of Neuroscience Research | 2011 |
| 303 | Song J, Lyu Y. Wang P. Nie Y. Lu H. Gao L. Tong X. | Treatment of naoxueshu promotes improvement of hematoma absorption and neurological function in acute intracerebral hemorrhage patients | Frontiers in phsiology | 2018 |
| 304 | Sprigg N, Flaherty K. Appleton J. P. Al-Shahi Salman R. Bereczki D. Beridze M. Christensen H. Ciccone A. Collins R. Czlonkowska A. Dineen R. A. Duley L. Egea-Guerrero J. J. England T. J. Krishnan K. Laska A. C. Law Z. K. Ozturk S. Pocock S. J. Roberts I. Robinson T. G. Roffe C. Seiffge D. Scutt P. Thanabalan J. Werring D. Whynes D. Bath P. M. Tich-Investigators | Tranexamic acid for hyperacute primary IntraCerebral Haemorrhage (TICH-2): an international randomised, placebo-controlled, phase 3 superiority trial | Lancet | 2018 |
| 305 | Sreekrishnan, Anirudh; Leasure, Audrey C.; Shi, Fu-Dong; Hwang, David Y.; Schindler, Joseph L.; Petersen, Nils H.; Gilmore, Emily J.; Kamel, Hooman; Sansing, Lauren H.; Greer, David M.; Sheth, Kevin N. | Functional Improvement Among Intracerebral Hemorrhage (ICH) Survivors up to 12 Months Post-injury | Neurocritical Care | 2017 |
| 306 | Staykov, Dimitre; Wagner, Ingrid; Volbers, Bastian; Doerfler, Arnd; Schwab, Stefan; Kollmar, Rainer | Mild prolonged hypothermia for large intracerebral hemorrhage | Neurocritical Care | 2013 |
| 307 | Steiner T, Poli S. Griebe M. Husing J. Hajda J. Freiberger A. Bendszus M. Bosel J. Christensen H. Dohmen C. Hennerici M. Kollmer J. Stetefeld H. Wartenberg K. E. Weimar C. Hacke W. Veltkamp R. | Fresh frozen plasma versus prothrombin complex concentrate in patients with intracranial haemorrhage related to vitamin K antagonists (INCH): a randomised trial | The Lancet: Neurology | 2016 |
| 308 | Su, W.; Gao, C.; Wang, P.; Huang, J.; Qian, Y.; Guo, L.; Zhang, J.; Jiang, R. | Correlation of Circulating T Lymphocytes and Intracranial Hypertension in Intracerebral Hemorrhage | World Neurosurgery | 2017 |
| 309 | Su, Xinhui; Zheng, Kunmu; Ma, Qilin; Huang, Jingxiong; He, Xiaojiang; Chen, Guibing; Wang, Weixing; Su, Fu; Tang, Hui; Wu, Hua; Tong, Suijun | Effect of local mild hypothermia on regional cerebral blood flow in patients with acute intracerebral hemorrhage assessed by 99mTc-ECD SPECT imaging | Journal of X-ray science and technology | 2015 |
| 310 | Su, Y. Y.; Wang, M.; Liu, Y. F.; Ye, H.; Gao, D. Q.; Chen, W. B.; Zhang, Y. Z.; Zhang, Y. | Module modified acute physiology and chronic health evaluation II: predicting the mortality of neuro-critical disease | Neurological Research | 2014 |
| 311 | Sun, D. T. F.; Tsang, Y. Y. Y.; Poon, W. S. | Intracerebral haematoma: Operative versus non-operative | Annals of the College of Surgeons of Hong Kong | 2004 |
| 312 | Sun, De-Biao; Xu, Meng-Jun; Chen, Qing-Meng; Hu, Hai-Tao | Significant elevation of serum caspase-3 levels in patients with intracerebral hemorrhage | International journal of clinical chemistry | 2017 |
| 313 | Sun, Haixin; Liu, Hongmei; Li, Di; Liu, Liping; Yang, Jun; Wang, Wenzhi | An effective treatment for cerebral hemorrhage: minimally invasive craniopuncture combined with urokinase infusion therapy | Neurological Research | 2010 |
| 314 | Sun, Y. M.; You, S. J.; Zhong, C. K.; Huang, Z. C.; Hu, L. F.; Zhang, X.; Shi, J. J.; Cao, Y. J.; Liu, C. F. | Neutrophil to lymphocyte ratio and the hematoma volume and stroke severity in acute intracerebral hemorrhage patients | American Journal of Emergency Medicine | 2017 |
| 315 | Sun, Y. Z.; Xu, B. Q.; Zhang, Q. | Nerve growth factor in combination with Oxiracetam in the treatment of Hypertensive Cerebral Hemorrhage | Pakistan Journal of Medical Sciences | 2018 |
| 316 | Suthar, Nilay N.; Patel, Khushali L.; Saparia, Chirag; Parikh, Ami P. | Study of clinical and radiological profile and outcome in patients of intracranial hemorrhage | Annals of African medicine | 2016 |
| 317 | Swor, Dionne E.; Thomas, Leena F.; Maas, Matthew B.; Grimaldi, Daniela; Manno, Edward M.; Sorond, Farzaneh A.; Batra, Ayush; Kim, Minjee; Prabhakaran, Shyam; Naidech, Andrew M.; Liotta, Eric M. | Admission Heart Rate Variability is Associated with Fever Development in Patients with Intracerebral Hemorrhage | Neurocritical Care | 2019 |
| 318 | Sykora, Marek; Diedler, Jennifer; Rupp, Andre; Turcani, Peter; Rocco, Andrea; Steiner, Thorsten | Impaired baroreflex sensitivity predicts outcome of acute intracerebral hemorrhage | Critical Care Medicine | 2008 |
| 319 | Szczudlik, Andrzej; Turaj, Wojciech; Slowik, Agnieszka; Strojny, Jacek | Hyperthermia is not an independent predictor of greater mortality in patients with primary intracerebral hemorrhage | Medical science monitor : international medical journal of experimental and clinical research | 2002 |
| 320 | Tan Sh, Ng P. Y. Yeo T. T. Wong S. H. Ong P. L. Venketasubramanian N. | Hypertensive basal ganglia hemorrhage: a prospective study comparing surgical and nonsurgical management | Surgical neurology | 2001 |
| 321 | Teleanu, D. M. | Decompressive craniectomy in deep spontaneous intracerebral hemorrhages | Romanian Journal of Neurology | 2014 |
| 322 | Terceno, Mikel; Serena, Joaquin; Bashir, Saima; Vera-Monge, Victor Augusto; Puig, Josep; Blasco, Gerard; Puig, Marc; Silva, Yolanda | Serotonin Reuptake Inhibitor Drugs Are Associated with Contrast Extravasation and Clinical Outcome in Patients with Intracerebral Haemorrhage | European neurology | 2019 |
| 323 | Tirschwell, David L.; Ton, Thanh G. N.; Ly, Kiet A.; Van Ngo, Quang; Vo, Tung T.; Pham, Chien Hung; Longstreth, William T., Jr.; Fitzpatrick, Annette L. | A prospective cohort study of stroke characteristics, care, and mortality in a hospital stroke registry in Vietnam | BMC Neurology | 2012 |
| 324 | Toscano, M.; Cecconi, E.; Capiluppi, E.; Vigano, A.; Bertora, P.; Campiglio, L.; Mariani, C.; Petolicchio, B.; D'Elia, T. S.; Verzina, A.; Vicenzini, E.; Fiorelli, M.; Cislaghi, G.; Di Piero, V. | Neuroanatomical, clinical and cognitive correlates of post-stroke dysphagia | European neurology | 2015 |
| 325 | Tsai, Y. H.; Hsu, L. M.; Weng, H. H.; Lee, M. H.; Yang, J. T.; Lin, C. P. | Voxel-based analysis of apparent diffusion coefficient in perihaematomal oedema: associated factors and outcome predictive value for intracerebral haemorrhage | BMJ Open | 2011 |
| 326 | Tsai, Y. H.; Hsu, L. M.; Weng, H. H.; Lee, M. H.; Yang, J. T.; Lin, C. P. | Functional diffusion map as an imaging predictor of functional outcome in patients with primary intracerebral haemorrhage | British Journal of Radiology | 2013 |
| 327 | Tsai, Yuan-Hsiung; Lee, Ming-Hsueh; Weng, Hsu-Huei; Chang, Sheng-Wei; Yang, Jen-Tsung; Huang, Yen-Chu | Fate of diffusion restricted lesions in acute intracerebral hemorrhage | PloS one | 2014 |
| 328 | Tsivgoulis, G.; Lioutas, V. A.; Varelas, P.; Katsanos, A. H.; Goyal, N.; Mikulik, R.; Barlinn, K.; Krogias, C.; Sharma, V. K.; Vadikolias, K.; Dardiotis, E.; Karapanayiotides, T.; Pappa, A.; Zompola, C.; Triantafyllou, S.; Kargiotis, O.; Ioakeimidis, M.; Giannopoulos, S.; Kerro, A.; Tsantes, A.; Mehta, C.; Jones, M.; Schroeder, C.; Norton, C.; Bonakis, A.; Chang, J.; Alexandrov, A. W.; Mitsias, P.; Alexandrov, A. V. | Direct oral anticoagulant- vs vitamin K antagonist-related nontraumatic intracerebral hemorrhage | Neurology | 2017 |
| 329 | Tuhrim, S.; Horowitz, D. R.; Sacher, M.; Godbold, J. H. | Volume of ventricular blood is an important determinant of outcome in supratentorial intracerebral hemorrhage | Critical Care Medicine | 1999 |
| 330 | Valiente, Raul Alberto; de Miranda-Alves, Maramelia Araujo; Silva, Gisele Sampaio; Gomes, Daniela Laranja; Brucki, Sonia Maria Dozzi; Rocha, Maria Sheila Guimaraes; Massaro, Ayrton Roberto | Clinical features associated with early hospital arrival after acute intracerebral hemorrhage: challenges for new trials | Cerebrovascular Diseases | 2008 |
| 331 | Venkatasubramanian, C.; Mlynash, M.; Finley-Caulfield, A.; Eyngorn, I.; Kalimuthu, R.; Snider, R. W.; Wijman, C. A. | Natural history of perihematomal edema after intracerebral hemorrhage measured by serial magnetic resonance imaging | Stroke | 2011 |
| 332 | Venkatasubramanian, Chitra; Kleinman, Jonathan T.; Fischbein, Nancy J.; Olivot, Jean-Marc; Gean, Alisa D.; Eyngorn, Irina; Snider, Ryan W.; Mlynash, Michael; Wijman, Christine A. C. | Natural history and prognostic value of corticospinal tract Wallerian degeneration in intracerebral hemorrhage | Journal of the American Heart Association | 2013 |
| 333 | Vespa P, Hanley D. Betz J. Hoffer A. Engh J. Carter R. Nakaji P. Ogilvy C. Jallo J. Selman W. Bistran-Hall A. Lane K. McBee N. Saver J. Thompson R. E. Martin N. Ices Investigators | ICES (Intraoperative Stereotactic Computed Tomography-Guided Endoscopic Surgery) for Brain Hemorrhage: a Multicenter Randomized Controlled Trial | Stroke | 2016 |
| 334 | Wada, R.; Aviv, R. I.; Fox, A. J.; Sahlas, D. J.; Gladstone, D. J.; Tomlinson, G.; Symons, S. P. | CT angiography "spot sign" predicts hematoma expansion in acute intracerebral hemorrhage | Stroke | 2007 |
| 335 | Wada, Shinichi; Yoshimura, Sohei; Inoue, Manabu; Matsuki, Takayuki; Arihiro, Shoji; Koga, Masatoshi; Kitazono, Takanari; Makino, Hisashi; Hosoda, Kiminori; Ihara, Masafumi; Toyoda, Kazunori | Outcome Prediction in Acute Stroke Patients by Continuous Glucose Monitoring | Journal of the American Heart Association | 2018 |
| 336 | Wang N, Liu H. Y. Zou Y. J. | Impact of the application of oxygenised liquid during the surgery on serous myelin basic protein in the patients with hypertensive cerebral haemorrhage | Chinese journal of clinical rehabilitation | 2004 |
| 337 | Wang, D. M.; Li, J.; Liu, J. R.; Hu, H. Y. | Diffusion Tensor Imaging Predicts Long-Term Motor Functional Outcome in Patients with Acute Supratentorial Intracranial Hemorrhage | Cerebrovascular Diseases | 2012 |
| 338 | Wang, Fei; Hu, Shanyou; Ding, Yong; Ju, Xuefeng; Wang, Li; Lu, Qiuxia; Wu, Xiao | Neutrophil-to-Lymphocyte Ratio and 30-Day Mortality in Patients with Acute Intracerebral Hemorrhage | Journal of stroke and cerebrovascular diseases | 2016 |
| 339 | Wang, Guo-Qiang; Li, Shi-Qiang; Huang, Yong-Hua; Zhang, Wei-Wei; Ruan, Wen-Wei; Qin, Jia-Zhen; Li, Ying; Yin, Wei-Min; Li, Yun-Jun; Ren, Zheng-Jun; Zhu, Ji-Qiang; Ding, Yun-Yan; Peng, Jun-Qi; Li, Pei-Jian | Can minimally invasive puncture and drainage for hypertensive spontaneous Basal Ganglia intracerebral hemorrhage improve patient outcome: a prospective non-randomized comparative study | Military Medical Research | 2014 |
| 340 | Wang, Hai-Qiao; Bao, Chun-Ling; Jiao, Zhi-Hua; Dong, Gui-Rong | Efficacy and safety of penetration acupuncture on head for acute intracerebral hemorrhage: A randomized controlled study | Medicine | 2016 |
| 341 | Wang, Ke; Du, Hang-Gen; Yin, Li-Chun; He, Min; Hao, Bi-Lie; Chen, Li | Which side of lateral ventricles to choose during external ventricular drainage in patients with intraventricular hemorrhage: ipsilateral or contralateral? | The Journal of Surgical Research | 2013 |
| 342 | Wang, Kuo-Wei; Cho, Chung-Lung; Chen, Han-Jung; Liang, Cheng-Loong; Liliang, Po-Chou; Tsai, Yu-Duan; Wang, Hao-Kuang; Lu, Kang | Molecular biomarker of inflammatory response is associated with rebleeding in spontaneous intracerebral hemorrhage | European neurology | 2011 |
| 343 | Wang, Lin-Guo; Huangfu, Xue-Qin; Tao, Bo; Zhong, Guan-Jin; Le, Zhou-Di | Serum tenascin-C predicts severity and outcome of acute intracerebral hemorrhage | Journal of clinical chemistry | 2018 |
| 344 | Wang, Qiuxiao; Wang, Deren; Liu, Ming; Fang, Yuan; You, Chao; Dong, Wei; Chang, Xueli; Lei, Chunyan; Zhang, Junhuai; Chen, Yanchao | Is diabetes a predictor of worse outcome for spontaneous intracerebral hemorrhage? | Clinical neurology and neurosurgery | 2015 |
| 345 | Wang, W. Z.; Jiang, B.; Liu, H. M.; Li, D.; Lu, C. Z.; Zhao, Y. D.; Sander, J. W. | Minimally invasive craniopuncture therapy vs. conservative treatment for spontaneous intracerebral hemorrhage: results from a randomized clinical trial in China | International Journal of stroke | 2009 |
| 346 | Wang, Wenjuan; Yang, Zhonghua; Liu, Liping; Dornbos, David, 3rd; Wang, Chunxue; Song, Xinjie; Gong, Xiping; Wang, Anxin; Zhao, Xingquan | Relationship between transcranial Doppler variables in acute stage and outcome of intracerebral hemorrhage | Neurocritical Care | 2011 |
| 347 | Wang, Z.; Qu, J.; Zhao, H. | Comparison of the clinical effects of stereotactic aspiration and craniotomies in the treatment of hypertensive intracerebral hemorrhages | International Journal of Clinical and Experimental Medicine | 2019 |
| 348 | Wayan, N.; Anne, S.; Tjokorda, M. | Combination Intracerebral Hemorrhage-graeb Score Improves Prediction of Outcome in Spontaneous Intracerebral Hemorrhage | Biomedical and Pharmacology Journal | 2019 |
| 349 | Wibawa, G. A.; Al Fauzi, A. | The relation of stroke patient's cortisol level to national institutes of health stroke scale core on spontaneous intracerebral hemorrhage: An observasional study at Rumah Sakit Umum Daerah (regional public hospital) Dr. Soetomo in Surabaya | Asian Journal of Pharmaceutical and Clinical Research | 2019 |
| 350 | Wu, Guofeng; Li, Shen; Wang, Likun; Mao, Yuanhong | The perihematomal glutamate level is associated with the outcome of patients with basal ganglia hematomas treated by minimally invasive procedures | Neurological Research | 2013 |
| 351 | Wu, Guofeng; Wang, Likun; Liu, Jian; Mao, Yuanhong; Qin, Guannan | Minimally invasive procedures reduced the damages to motor function in patients with thalamic hematoma: observed by motor evoked potential and diffusion tensor imaging | Journal of stroke and cerebrovascular diseases : the official journal of National Stroke Association | 2013 |
| 352 | Xia, Chao; Lin, Sen; Yang, Jie; He, Sha; Li, Hao; Liu, Ming; You, Chao | Genetic Variations of COL4A1 Gene and Intracerebral Hemorrhage Outcome: A Cohort Study in a Chinese Han Population | World Neurosurgery | 2018 |
| 353 | Xiao, Bo; Wu, Fang-Fang; Zhang, Hong; Ma, Yan-Bin | A randomized study of urgent computed tomography-based hematoma puncture and aspiration in the emergency department and subsequent evacuation using craniectomy versus craniectomy only | Journal of Neurosurgery | 2012 |
| 354 | Xiong, L.; Yang, Y.; Zhang, M.; Xu, W. | The use of serum glial fibrillary acidic protein test as a promising tool for intracerebral hemorrhage diagnosis in Chinese patients and prediction of the short-term functional outcomes | Neurological Sciences | 2015 |
| 355 | Xu, Q.; Wei, Y. T.; Fan, S. B.; Wang, L.; Zhou, X. P. | Repetitive hyperbaric oxygen treatment increases insulin sensitivity in diabetes patients with acute intracerebral hemorrhage | Neuropsychiatric Disease and Treatment | 2017 |
| 356 | Yadav, Yad Ram; Mukerji, Gaurav; Shenoy, Ravikiran; Basoor, Abhijeet; Jain, Gaurav; Nelson, Adam | Endoscopic management of hypertensive intraventricular haemorrhage with obstructive hydrocephalus | BMC Neurology | 2007 |
| 357 | Yan, Xin-Jiang; Yu, Guo-Feng; Jie, Yuan-Qing; Fan, Xiao-Feng; Huang, Qiang; Dai, Wei-Min | Role of galectin-3 in plasma as a predictive biomarker of outcome after acute intracerebral hemorrhage | Journal of neurological sciences | 2016 |
| 358 | Yang, Kang; Feng, Yulan; Mu, JinJin; Fu, Ningzhen; Chen, Shufen; Fu, Yi | The Presence of Previous Cerebral Microbleeds Has a Negative Effect on Hypertensive Intracerebral Hemorrhage Recovery | Frontiers in aging neuroscience | 2017 |
| 359 | Yang, Xiaobo; Ren, Weimin; Zu, Hengbing; Dong, Qiang | Evaluate the serum cortisol in patients with intracerebral hemorrhage | Clinical neurology and neurosurgery | 2014 |
| 360 | Yang, Y. L.; Zhang, L. L.; He, X.; Zhou, Y. M.; Chen, G. Q.; Xu, M.; Zhou, J. X. | Use of the Bispectral Index to Predict Recovery of Consciousness in Patients with Spontaneous Intracerebral Hemorrhage After Surgical Hematoma Evacuation: A Prospective Cohort Study | Medical Science Monitor | 2019 |
| 361 | Ye, Z. P.; Ai, X. L.; Zheng, J.; Ma, L.; Lin, S.; You, C.; Li, H. | The Effect of Cerebrovascular Stenosis on Peri-Hematoma Cerebral Perfusion and Clinical Outcomes in Patients with Supratentorial Spontaneous Intracerebral Hemorrhage | Medical Science Monitor | 2018 |
| 362 | Yelnik, A. P.; Quintaine, V.; Andriantsifanetra, C.; Wannepain, M.; Reiner, P.; Marnef, H.; Evrard, M.; Meseguer, E.; Devailly, J. P.; Lozano, M.; Lamy, C.; Colle, F.; Vicaut, E. | AMOBES (Active Mobility Very Early after Stroke): A Randomized Controlled Trial | Stroke | 2017 |
| 363 | Yoshioka, Hideyuki; Horikoshi, Toru; Aoki, Shigeki; Hori, Masaaki; Ishigame, Keiichi; Uchida, Mikito; Sugita, Masao; Araki, Tsutomu; Kinouchi, Hiroyuki | Diffusion tensor tractography predicts motor functional outcome in patients with spontaneous intracerebral hemorrhage | Neurosurgery | 2008 |
| 364 | You, Shoujiang; Shi, Luyao; Zhong, Chongke; Xu, Jiaping; Han, Qiao; Zhang, Xia; Liu, Huihui; Zhang, Yanlin; Shi, Jijun; Huang, Zhichao; Cao, Yongjun; Liu, Chunfeng | Prognostic Significance of Estimated Glomerular Filtration Rate and Cystatin C in Patients with Acute Intracerebral Hemorrhage | Cerebrovascular Diseases | 2016 |
| 365 | Young, Z. K.; Kyu, H. K. | Even in patients with a small hemorrhagic volume, stereotactic-guided evacuation of spontaneous intracerebral hemorrhage improves functional outcome | Journal of Korean Neurosurgical Society | 2009 |
| 366 | Yu M, Zhang D. P. Ren Y. F. Zhang H. T. Fu S. Q. Zhang S. L. | Clinical efficacy and safety of edaravone therapy in acute cerebral haemorrhage | Tropical Journal of Pharmaceutical Research | 2016 |
| 367 | Yu Y, Zhao W. Zhu C. Kong Z. Xu Y. Liu G. Gao X. | The clinical effect of deferoxamine mesylate on edema after intracerebral hemorrhage | PloS one | 2015 |
| 368 | Yu Yl, Kumana C. R. Lauder I. J. Cheung Y. K. Chan F. L. Kou M. Chang C. M. Cheung R. T. Fong K. Y. | Treatment of acute cerebral hemorrhage with intravenous glycerol. A double-blind, placebo-controlled, randomized trial | Stroke | 1992 |
| 369 | Yu, W. H.; Wang, W. H.; Dong, X. Q.; Du, Q.; Yang, D. B.; Shen, Y. F.; Wang, H.; Zhang, Z. Y.; Zhu, Q.; Che, Z. H.; Liu, Q. J.; Jiang, L.; Du, Y. F. | Prognostic significance of plasma copeptin detection compared with multiple biomarkers in intracerebral hemorrhage | Clinica Chimica Acta | 2014 |
| 370 | Yuan, Ruozhen; Wang, Deren; Liu, Ming; Liu, Junfeng; He, Yongqiao; Deng, Yongyi; Lei, Chunyan; Hao, Zilong; Tao, Wendan; Liu, Bian; Chang, Xueli; Wang, Qiuxiao; Tan, Ge | Long-Term Prognosis of Spontaneous Intracerebral Hemorrhage on the Tibetan Plateau: A Prospective Cohort Study at 2 Hospitals | World Neurosurgery | 2016 |
| 371 | Zang, Y. J.; Zhang, C. H.; Song, Q.; Zhang, J.; Li, H. X.; Zhang, C. L.; Feng, S. S.; Gu, F. | Therapeutic effect of early intensive antihypertensive treatment on rebleeding and perihematomal edema in acute intracerebral hemorrhage | Journal of clinical hypertension | 2019 |
| 372 | Zeng, Jingsong; Zheng, Ping; Tong, Wusong; Fang, Weimin | Decreased risk of secondary brain herniation with intracranial pressure monitoring in patients with haemorrhagic stroke | BMC anesthesiology | 2014 |
| 373 | Zeng, L. L.; Tang, G. H.; Wang, J.; Zhong, J. B.; Xia, Z. Y.; Li, J. X.; Chen, G. S.; Zhang, Y. B.; Luo, S. H.; Huang, G.; Zhao, Q. S.; Wan, Y.; Chen, C. J.; Zhu, K. Y.; Qiao, H. Z.; Huang, T.; Liu, X.; Zhang, Q. X.; Lin, R. M.; Li, H. J.; Gong, B. Y.; Chen, X. Y.; Zhou, Y. X.; Wen, Z. H.; Guo, J. W. | Safety and efficacy of herbal medicine for acute intracerebral hemorrhage (CRRICH): a multicentre randomised controlled trial | BMJ Open | 2019 |
| 374 | Zhang, Huan-Xin; Fan, Qun-Xiong; Xue, Shi-Zhen; Zhang, Min; Zhao, Ji-Xian | Twenty-four-hour blood pressure variability plays a detrimental role in the neurological outcome of hemorrhagic stroke | The Journal of international medical research | 2018 |
| 375 | Zhang, Jixin; Lu, Shiyong; Wang, Suzhen; Zhou, Naiyun; Li, Guoliang | Comparison and analysis of the efficacy and safety of minimally invasive surgery and craniotomy in the treatment of hypertensive intracerebral hemorrhage | Pakistan Journal of Medical Sciences | 2018 |
| 376 | Zhang, P.; Huang, H.; Chen, F. J. | EFFECT OF NIMODIPINE INJECTION ON THE CURATIVE EFFECT, OVERALL PROGNOSIS AND LEVEL OF SERUM IL-6, AND TNF-A IN PATIENTS WITH HYPERTENSIVE INTRACEREBRAL HAEMORRHAGE | Acta Medica Mediterranea | 2019 |
| 377 | Zhang, Xin; Lu, Xiao-Min; Huang, Li-Fa; Li, Xu | Prognostic value of leptin: 6-month outcome in patients with intracerebral hemorrhage | Peptides | 2013 |
| 378 | Zhang, Y. H.; Al-Aref, R.; Fu, H. X.; Yang, Y. X.; Feng, Y. G.; Zhao, C.; Dong, J.; Sun, G. Q. | Neuronavigation-Assisted Aspiration and Electro-Acupuncture for Hypertensive Putaminal Hemorrhage: A Suitable Technique on Hemiplegia Rehabilitation | Turkish Neurosurgery | 2017 |
| 379 | Zhang, Y. H.; Ding, W.; Yang, Y. X.; Xu, H. C.; Xiong, F.; Liu, C. Z. | Effects of Transsylvian-Transinsular Approach to Hypertensive Putaminal Hematoma Operation and Electroacupuncture on Motor Recovery | Journal of Craniofacial Surgery | 2011 |
| 380 | Zhang, Yi-Bin; Zheng, Shu-Fa; Yao, Pei-Sen; Chen, Guo-Rong; Li, Guang-Hai; Li, Song-Chuan; Zheng, Yi-Fang; Wang, Jian-Qun; Kang, De-Zhi; Shang-Guan, Huang-Cheng | Lower Ionized Calcium Predicts Hematoma Expansion and Poor Outcome in Patients with Hypertensive Intracerebral Hemorrhage | World Neurosurgery | 2018 |
| 381 | Zhang, Z. L.; Liu, Y. G.; Huang, Q. B.; Wang, H. W.; Song, Y.; Xu, Z. K.; Li, F. | Nuclear factor-kappaB activation in perihematomal brain tissue correlates with outcome in patients with intracerebral hemorrhage | Journal of Neuroinflammation | 2015 |
| 382 | Zhang, Zaiqiang; Li, Xingang; Liu, Yuguang; Shao, Yi; Xu, Shujun; Yang, Yang | Application of neuroendoscopy in the treatment of intraventricular hemorrhage | Cerebrovascular Diseases | 2007 |
| 383 | Zhao, J. Z.; Zhou, L. F.; Zhou, D. B.; Wang, R. Z.; Wang, M.; Wang, D. J.; Wang, S.; Yuan, G.; Kang, S.; Ji, N.; Zhao, Y. L.; Ye, X. | Computed tomography-guided aspiration versus key-hole craniotomy for spontaneous putaminal haemorrhage: a prospective comparison of minimally invasive procedures | Hong Kong Medical Journal | 2009 |
| 384 | Zhao, Jianhua; Mao, Qing; Qian, Zhongxin; Zhu, Jun; Qu, Zhun; Wang, Chao | Effect of mild hypothermia on expression of inflammatory factors in surrounding tissue after minimally invasive hematoma evacuation in the treatment of hypertensive intracerebral hemorrhage | Experimental and therapeutic medicine | 2018 |
| 385 | Zhao, Yanxia; Yang, Jie; Zhao, Hongdong; Ding, Yunlong; Zhou, Junshan; Zhang, Yingdong | The association between hyperglycemia and the prognosis of acute spontaneous intracerebral hemorrhage | Neurological Research | 2017 |
| 386 | Zhao, Z. Y.; Wang, H. Y.; Li, Z.; Wang, X. X.; Zhang, W.; Feng, K.; Fan, J. C. | Assessment of the effect of short-term factors on surgical treatments for hypertensive intracerebral haemorrhage | Clinical neurology and neurosurgery | 2016 |
| 387 | Zheng J, Li H. Lin S. Ma J. Guo R. Ma L. Fang Y. Tian M. Liu M. You C. | Perioperative Antihypertensive Treatment in Patients With Spontaneous Intracerebral Hemorrhage | Stroke | 2017 |
| 388 | Zheng, Guan-Rong; Chen, Bin; Shen, Jia; Qiu, Shen-Zhong; Yin, Huai-Ming; Mao, Wei; Wang, Hong-Xiang; Gao, Jian-Bo | Serum myeloperoxidase concentrations for outcome prediction in acute intracerebral hemorrhage | International journal of clinical chemistry | 2018 |
| 389 | Zheng, T.; Zhu, X.; Liang, H.; Huang, H.; Yang, J.; Wang, S. | Impact of early enteral nutrition on short term prognosis after acute stroke | Journal of Clinical Neuroscience | 2015 |
| 390 | Zhou, Houguang; Zhang, Yu; Liu, Ling; Han, Xu; Tao, Yinghong; Tang, Yuping; Hua, Wei; Xue, Jianzhong; Dong, Qiang | A prospective controlled study: minimally invasive stereotactic puncture therapy versus conventional craniotomy in the treatment of acute intracerebral hemorrhage | BMC Neurology | 2011 |
| 391 | Zidverc-Trajkovic, J.; Kovacevic, M. S.; Jovanovic, D.; Beslac-Bumbasirevic, L.; Bugarski-Prokopljevic, C. | Headache as a first symptom of non-traumatic intracerebral hemorrhage | Headache Quarterly | 1998 |
| 392 | Zorzon, M.; Mase, G.; Biasutti, E.; Vitrani, B.; Cazzato, G. | Predictors of long-term case fatality (one-year) in primary intracerebral hemorrhage | Europa Medicophysica | 1999 |
| 393 | Zuccarello, M.; Brott, T.; Derex, L.; Kothari, R.; Sauerbeck, L.; Tew, J.; Van Loveren, H.; Yeh, H. S.; Tomsick, T.; Pancioli, A.; Khoury, J.; Broderick, J. | Early surgical treatment for supratentorial intracerebral hemorrhage - A randomized feasibility study | Stroke | 1999 |
| 394 | Zuo, Yi; Cheng, Gang; Gao, Da-Kuan; Zhang, Xiang; Zhen, Hai-Ning; Zhang, Wei; Xiao, San-Chao | Gross-total hematoma removal of hypertensive basal ganglia hemorrhages: a long-term follow-up | Journal of neurological sciences | 2009 |
| 395 | Zweifel, Christian; Katan, Mira; Schuetz, Philipp; Ernst, Andrea; Mariani, Luigi; Muller, Beat; Christ-Crain, Mirjam | Growth hormone and outcome in patients with intracerebral hemorrhage: a pilot study | Biomarkers | 2011 |
