# Supplementary material for: How outcomes are measured after spontaneous intracerebral hemorrhage: A systematic scoping review
Source: PLoS One. 2021 Jun 30;16(6):e0253964. doi: 10.1371/journal.pone.0253964 (PMC8244847; doi:10.1371/journal.pone.0253964)
Supplement: S2 Table — (DOCX) [file pone.0253964.s003.docx]

SUPPLEMENTAL MATERIAL

How Outcomes are Measured After Spontaneous Intracerebral Hemorrhage: A Systematic Scoping Review of the Current Prospective Literature

S2 Table. Outcomes reported in 2 or fewer studies.

| Reported Outcome | Number of assessments |
| --- | --- |
| Chinese Stroke Scale | 2 |
| EQ-5D-3L | 2 |
| European Stroke Scale | 2 |
| Modified Rankin Scale (5 points) | 2 |
| Montreal Cognitive Assessment (MoCA) | 2 |
| Quality of Life in Neurological Disorders (NEURO-QOL) | 2 |
| Rappaport Disability Rating Scale | 2 |
| Short Form Health Survey (SF-36) | 2 |
| Swallow Function Assessment | 2 |
| ABILHAND manual ability measure | 1 |
| ADLBI | 1 |
| Aphasia severity rating scale | 1 |
| Canadian Neurological Scale | 1 |
| Canadian Stroke Scale | 1 |
| Clinical Neurological Function Deficit Scale (CNFDS) | 1 |
| Columbia Suicide Severity Rating Scale | 1 |
| EQ-5D-HSUV | 1 |
| EQ-VAS | 1 |
| Fatigue assessment scale | 1 |
| Functional Cognitive Index | 1 |
| General Health Questionnaire (GHQ-12) | 1 |
| Glasgow prognosis scale | 1 |
| Hamilton Depression Rating Scale | 1 |
| Hospital Anxiety and Depression Scale | 1 |
| Informant Questionnaire on Cognitive Decline in the Elderly (IQCODE) | 1 |
| Karnofsky score | 1 |
| Medical Research Council Scale | 1 |
| Modified Ashworth Scale | 1 |
| Modified Canadian Neurological Score | 1 |
| Modified Cognitive Ability Scale | 1 |
| Modified Edinburgh-Scandinavian Stroke Scale | 1 |
| Modified Glasgow Coma Scale | 1 |
| Modified Motor Assessment Scale | 1 |
| Modified NIHSS | 1 |
| Modified Telephone Mini-Mental State Examination (MMSE) | 1 |
| Module Modified - APACHE II | 1 |
| Motricity Index | 1 |
| MSK-SSP Manikin | 1 |
| Navier Stroke Scale | 1 |
| Nottingham Extended Activities of Daily Living | 1 |
| Postural Assessment Scale for stroke | 1 |
| Prognosis-based Barthel Index | 1 |
| Prognosis-based Modified Rankin Scale | 1 |
| PROMISE | 1 |
| Revised Hamilton Rating Scale for Depression | 1 |
| Scandinavian Stroke Study Group | 1 |
| ScreeLing | 1 |
| Short PortaBLE Mental Status Questionnaire (SPMSQ) | 1 |
| Simplified Fugl-Meyer Assessment Scale | 1 |
| Stroke Adapted Sickness Inventory Profile (SA-SIP30) | 1 |
| Traditional Chinese Medicine (TCM) score | 1 |
| Zung Self-Rated Anxiety Scale | 1 |
