# Supplementary material for: How outcomes are measured after spontaneous intracerebral hemorrhage: A systematic scoping review
Source: PLoS One. 2021 Jun 30;16(6):e0253964. doi: 10.1371/journal.pone.0253964 (PMC8244847; doi:10.1371/journal.pone.0253964)
Supplement: S1 File — (DOCX) [file pone.0253964.s004.docx]

SUPPLEMENTAL MATERIAL

How Outcomes are Measured After Spontaneous Intracerebral Hemorrhage: A Systematic Scoping Review of the Current Prospective Literature

**Supplemental Methods**

Search strategies from the date of inception to November 2019: MEDLINE, Embase, Cochrane Central Register of Controlled Trials, and Web of Science.

Database: Ovid MEDLINE(R) ALL <1946 to November 04, 2019>
Search Strategy:
--------------------------------------------------------------------------------
1     exp Cerebral Hemorrhage/dt, su, th [Drug Therapy, Surgery, Therapy]
2     Intracranial Hemorrhages/dt, su, th [Drug Therapy, Surgery, Therapy]
3     ((brain or intracerebral or cerebral) adj2 (hemorrhag* or haemorrhag*)).tw.
4     (brain h?emorrhag* or intracerebral h?emorrhag* or cerebral h?emorrhag*).kw.
5     h?emorrhag* stroke*.tw,kw.
6     or/1-5
7     Prospective Studies/
8     prospective.tw.
9     Observational Study/ or observational.tw.
10     randomized controlled trial.pt.
11     controlled clinical trial.pt.
12     randomi?ed.ab.
13     clinical trials as topic.sh.
14     randomly.ab.
15     trial.ti.
16     or/7-15
17     6 and 16
18     "Recovery of Function"/
19     "Outcome Assessment (Health Care)"/
20     outcome*.ti.
21     prognos*.mp.
22     survival rate/
23     functional outcome*.tw.
24     outcome measure*.tw.
25     (scor* or valid*).tw.
26     mortality/ or mortality.tw.
27     or/18-26
28     17 and 27
29     case reports.pt.
30     28 not 29
31     (child/ or infant/) not adult/
32     30 not 31
33     animals/ not humans/
34     32 not 33

Database: Embase Classic+Embase <1947 to 2019 November 04>
Search Strategy:
--------------------------------------------------------------------------------
1     brain hemorrhage/dt, su, th [Drug Therapy, Surgery, Therapy]
2     ((brain or intracerebral or cerebral) adj2 (hemorrhag* or haemorrhag*)).tw.
3     h?emorrhag* stroke*.tw.
4     or/1-3
5     prospective study/
6     prospective*.tw.
7     observational study/
8     observational.tw.
9     crossover-procedure/
10     double-blind procedure/
11     randomized controlled trial/
12     (random* or (doubl* adj blind*) or (singl* adj blind*)).tw.
13     or/5-12
14     4 and 13
15     *outcome assessment/
16     outcome*.ti.
17     prognos*.mp.
18     *functional status/
19     functional outcome*.tw.
20     outcome measure*.tw.
21     (scor* or valid*).tw.
22     *mortality/
23     mortality.tw.
24     survival rate/
25     or/15-24
26     14 and 25
27     case report/
28     26 not 27
29     (animal experiment/ or exp animals/) not human/
30     28 not 29
31     (infant/ or newborn/ or child/) not adult/
32     30 not 31

Database: EBM Reviews - Cochrane Central Register of Controlled Trials <September 2019>
Search Strategy:
--------------------------------------------------------------------------------
1     exp Cerebral Hemorrhage/
2     Intracranial Hemorrhages/
3     ((brain or intracerebral or cerebral) adj2 (hemorrhag* or haemorrhag*)).ti,ab.
4     h?emorrhag* stroke*.ti,ab.
5     1 or 2 or 3 or 4
6     "Recovery of Function"/
7     "Outcome Assessment (Health Care)"/
8     outcome*.ti.
9     prognos*.mp.
10     survival rate/
11     functional outcome*.tw.
12     outcome measure*.tw.
13     (scor* or valid*).tw.
14     mortality/ or mortality.tw.
15     or/6-14 (427055)
16     5 and 15 (2412)
17     (child or infant or newborn).hw.
18     adult.hw.
19     17 not 18
**20     16 not 19 (2292)**

Web of Science

1     TS=("cerebral hemorrhage" OR "intracranial hemorrhage*" OR "intracerebral h?emorrhag*" OR "h?emorrhag* stroke*"OR "brain h?emorrhag*")

2     TS=(prognos* OR survival OR mortality OR "functional outcome*" OR "outcome measure*" OR "recovery of function" OR scor* OR valid*)
3     TI=outcome*
4     #3 OR #2
5     #4 AND #1
6     TS= clinical trial* OR TS=research design OR TS=controlled trial* OR TS=observational OR TS=prospective* OR TS=random* OR TS=(single blind*) OR TS=(double blind*)
7     #6 AND #5
